# Design of a robust neural network-based controller for frequency stability in microgrids

**DOI:** 10.1038/s41598-026-52103-8

**Published:** 2026-05-25

**Authors:** Montaser Abdelsattar, Ibrahim A. Khalaf, Alaaeldien Hassan, Asmaa G. Ameen

**Affiliations:** 1 Electrical Engineering Department, Faculty of Engineering, Qena University, Qena, 83523 Egypt; 2 Artificial Intelligence Department, Faculty of Computers and Artificial Intelligence, Hurghada University, Hurghada, 84511 Egypt

**Keywords:** Microgrids, Virtual inertia, Proportional-integral-derivative-acceleration controller, Multi-layer feedforward neural network, Frequency stability, Energy science and technology, Engineering

## Abstract

The utilization of renewable energy sources (RESs), including solar and wind, in microgrids (MGs) present a critical challenge for maintaining system stability, mostly because of the elimination of mechanical inertia traditionally provided by synchronous generators. This study presents a controlling technique to guard disturbances in the islanded MG. To address this challenge, a multi-layer feedforward neural network (MLFFNN) is used to enhance the frequency stability of an islanded MG. The MLFFNN controller is compared to traditional controllers such as proportional-integral-derivative-acceleration (PIDA), proportional-integral-derivative (PID) and virtual inertia (VI) to evaluate its performance and effectiveness. Three scenarios are studied: load variations, RES fluctuations, and a combined case including both load variations and RES variability. A comparative study between VI, PID, PIDA, and MLFFNN controllers has been carried out, and shows that the MLFFNN combined with the VI controller is better than VI, PID, PIDA and MLFFNN controllers in all cases. Compared with the uncontrolled system, the MLFFNN with VI reduced the maximum frequency deviation from 4.92 to 3.86×$\:{10}^{-5}$ Hz in the third case. Finally, the MLFFNN combined with the VI controller provided better performance for frequency stability.

## Introduction

### Background and literature review

The increasing use of clean energy sources, particularly solar and wind power, is causing a rapid evolution of modern power systems^1,2^. However, these sources are sustainable for the environment^3,4^. They present serious challenges to grid stability due to their intermittent and non-inertial nature^5,6^. When generation and demand don’t match, an imbalance occurs^7^. The unbalanced system introduces frequency and voltage deviations, which cause a system’s decreased resilience and dependability. It can lead to complete blackouts or system collapses^8,9^. Due to the highly variable nature of renewable energy sources (RES), the low-inertia nature of these microgrids (MGs) presents problems with dynamic stability, like transient power impacts and unacceptable frequency changes^10,11^. Traditionally, frequency control has relied on the physical inertia of synchronous generators^12^. However, the displacement of these machines by inverter-based renewable sources significantly reduces the grid’s natural inertia^13^. To deal with this issue, the concept of virtual inertia (VI) was created, which simulates the inertial response with control algorithms and powered electronic converters^14^. The worldwide network is typically connected to MG, but in the event of an emergency, when significant disruptions occur, they cut off from the global network and can only supply significant local loads^15,16^. MG is a very important and necessary part of the development of the smart grid. An MG consists of loads, distributed energy storage devices, and distributed energy resources (DERs)^15^. DER devices can be charged with the power excess and discharge to compensate for the power deficit.

**Fig. 1:**
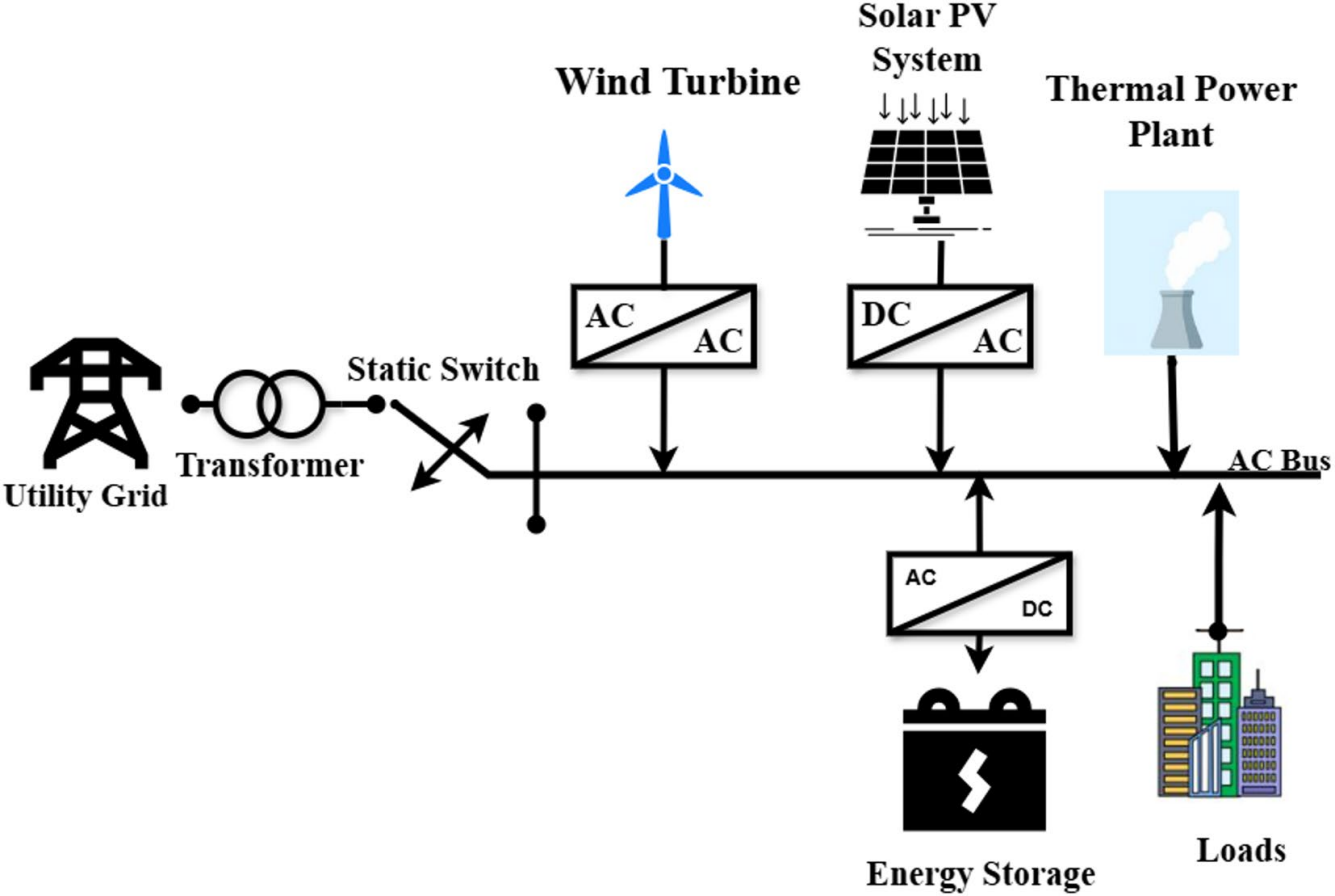
Structure of microgrid. Generated using [draw.io] v29.6.10.

**Fig. 2:**
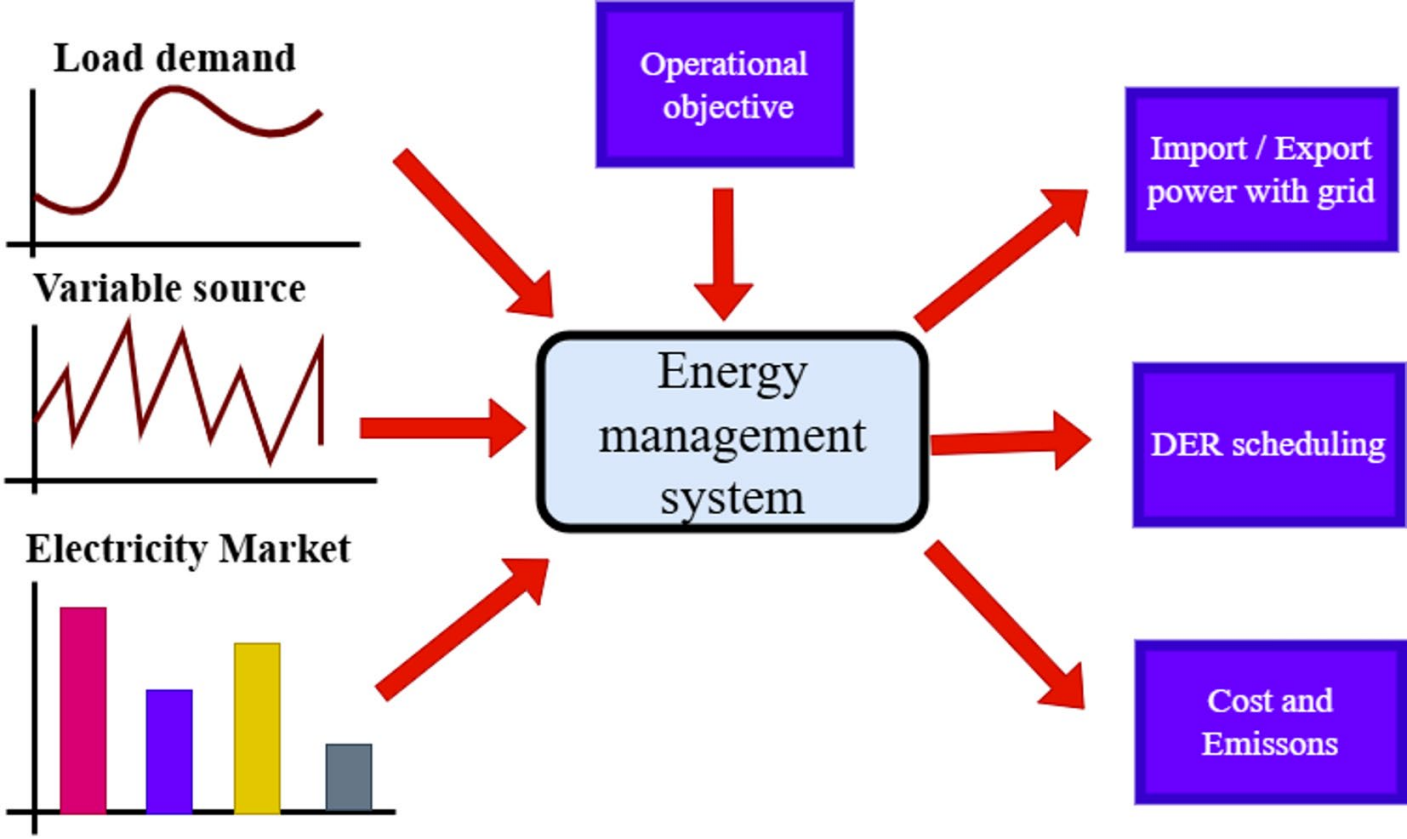
The energy management system. Generated using [draw.io] v29.6.10.

Thus, they contribute to the MGs increased dependability as well as its efficiency and economy^17^. Moreover, rapid reaction devices are another name for energy storage. Figure 1 illustrates a typical MG system comprising multiple components, including RESs (wind turbine and solar photovoltaics (PVs) system), a thermal power plant, an energy storage system, and loads, all interconnected through an AC bus^18^. MG is linked to the upstream network in the grid-connected mode^19^. Depending on power-sharing, the MG may receive all or a portion of the energy from the main grid^20^. However, when total production surpasses consumption, the excess electricity can be sent to the main grid. MG can seamlessly transition to islanded operation in the event of an upstream network outage or planned operations As a result, the MG functions independently, a feature known as island mode, much like the current islands’ electrical power systems^21^. It is necessary to have an energy management system (EMS) which is expected to optimize the power-sharing among DER, the cost of energy production and emissions. The functions of the proposed EMS are shown in Fig. 2. EMS receives the forecast values of load demand, the DER and the market electricity price in each hour on the next day to impose the cost, emissions, import/export electricity on the main grid, and the DER’s scheduled output power.

Since generators and motors at power plants and industrial sites rotate synchronously with the grid frequency, and their stored kinetic energy provides inertia to the electric power system^5^. This acts as a strong barrier against abrupt change. The grid’s frequency tends to decline when power consumption spikes. The grid’s large spinning mass reduces the rate of change of frequency and acts as a shock absorber for power fluctuations^22,23^. Solar, on the other hand, is grid-connected but lacks rotational mass. As they are not directly connected to the grid, even enormous wind turbines are unable to offer the required stability. Rather, a frequency converter device sits between the wind turbine and the power grid and stops the rotating mass’s kinetic energy from operating as inertia when the frequency varies. It becomes more challenging to maintain the frequency within its typical range of variation when inertia diminishes as abrupt frequency variations brought on by changes in power generation or consumption are quicker and more pronounced.

Furthermore, using VI improves the system’s power quality and reliability during disturbances. Virtual inertia control (VIC) can deliver uninterruptible power to MGs, enhancing system stability^24,25^. The problem of low inertia has been addressed using a number of methods. VI is one of the main approaches used in power systems to create more inertia. Power systems can improve their operational control by increasing their inertia with the help of VI. By increasing the MG’s inertia using VI simulation, RES penetration can increase.

Authors in^26^ presented an efficient frequency regulation approach for islanded MGs that use a fractional-order PID (FOPID) controller to enhance the frequency stability. Unlike the conventional proportional-integral-derivative (PID), which uses integer-order integration and differentiation, the FOPID includes two additional tuning parameters to fine-tune system dynamics. When compared to conventional PID controllers, the proposed one is more resilient to model uncertainty and load delays while preserving higher reliability margins. Additionally, the FOPID is compared to the strong H∞ controller. The results reveal that the FOPID provides comparable durability while having a simpler form and lower computing cost. A VIC technique based on an adaptive neuro-fuzzy inference system was proposed by the author in^27^. The controller of the Adaptive Neuro-Fuzzy Inference System was trained to adjust the damping coefficients and VI adaptively in response to disturbances and system conditions of operation. Although the ANFIS-based VIC showed good frequency stabilization, measurement delays and uncertainty in system parameters continue to present difficulties. In^28^ a proposed VI scheduling for MG is used to enhance both economy and security.

In^22^ authors suggested a cascaded control framework that combined three degree of freedom with frictional order controllers to enhance the frequency stability, the controller parameters were adjusted using the squirrel search algorithm but still depended on offline tuning which limited it’s adaptability under real time changes. A robust H∞- based VIC was suggested in^29^ to overcome the parameters uncertainty and the negative effects of phase locked loop measurement delays. Despite demonstrating strong robustness, its design complexity and limited flexibility. In^10^, the VI is improved by utilizing a differential evolution optimization approach. The system performance was assessed in three different scenarios: constant, unit-step, and random variation in load and RES output. The differential evolution technique was utilized to improve the controller settings. Even while the VIC-based differential evolution improved frequency stability, its restricted adaptability and reliance on storage devices stand out. Improved particle swarm optimization and genetic algorithm used in^30^ to enhance the frequency stability. But it depended on the population size. The algorithm may converge prematurely to a local optimum in small population size, the search becomes more accurate but computationally expensive when the population size is large. In order to overcome the weak inertia problem of an island MG with RES penetration, this study employed a VIC loop to enhance frequency in MG. Different signals (continuous, unit, and random step) were used to illustrate a variety of load/RES scenarios (wind and solar PV resources).

A comparison with a traditional a PID-VI, VI and without VI was carried out. The suggested controller produced more effective frequency regulation with a lower steady-state error and settling time. An additional comparison with^10^ put out in earlier research was done. The model of neural network schemes has three inputs: load variation, solar power and wind power, while the output is the frequency deviation of MG. A a multi-layer feedforward neural network (MLFFNN) is employed based on multi-input single-output modeling to mitigate the frequency deviation.

### Novelty and contributions

Compared with the existing literature, the main novelties and contributions of this work are as follows:

- A hybrid MLFFNN-based VI control strategy is proposed for improving frequency regulation in an islanded MG with high penetration of RESs.
- The proposed controller adopts a structure in which the MLFFNN generates an adaptive control signal that is processed through the VI block to provide synthetic inertial support during load and renewable power disturbances.
- Unlike standalone VI control, the proposed controller scheme combines the fast-transient support of virtual inertia with the nonlinear learning and adaptive capability of the MLFFNN, leading to enhanced dynamic frequency performance.
- A comparative analysis is carried out among without controller, VI, PID-VI, proportional-integral-derivative-acceleration (PIDA)-VI, MLFFNN, and MLFFNN + VI, demonstrating the effectiveness of the proposed hybrid controller in reducing the maximum frequency deviation and improving frequency stabilization.
- A comparative assessment of the proposed MLFFNN-VI controller against several conventional and literature-reported controllers.

**Fig. 3:**
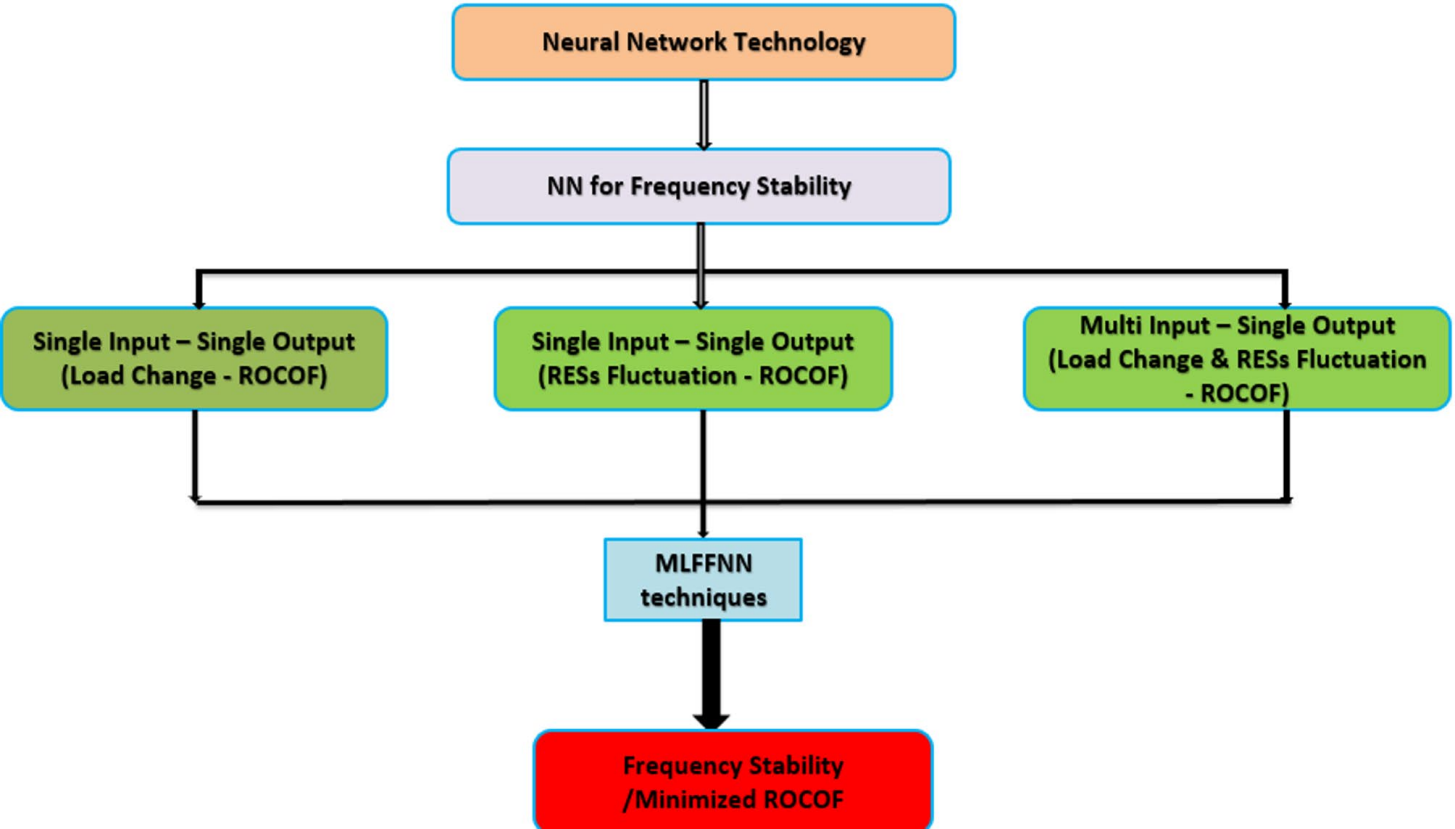
Neural network schemes for frequency stability improvement in microgrids using MLFFNN technique.

The proposed method for improving MG frequency stability using neural network technology is illustrated in Fig. 3 in this paper. Section  2 describes the MG model. Section  3 presents VI technique and control methodology. In Sect.  4, the results extracted from the simulations will be discussed, and finally, the conclusion will be presented in the last section.

## The system model

**Fig. 4:**
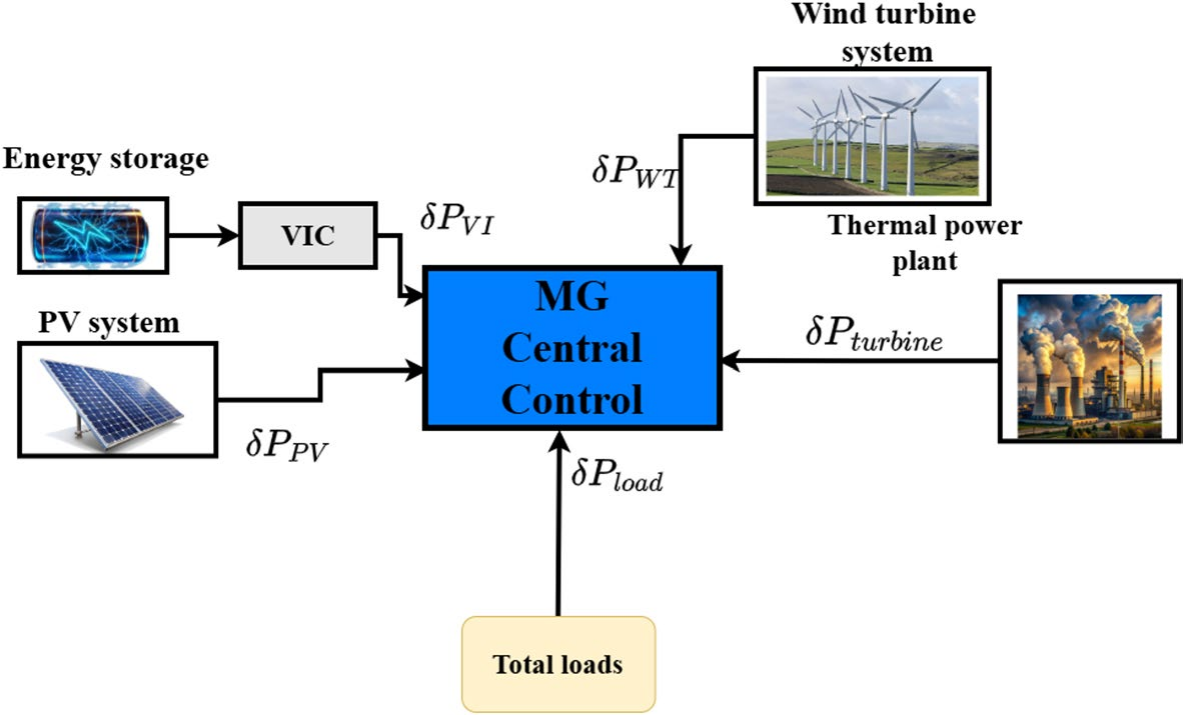
Scheme of islanded MG system. Generated using [draw.io] v29.6.10.

**Fig. 5:**
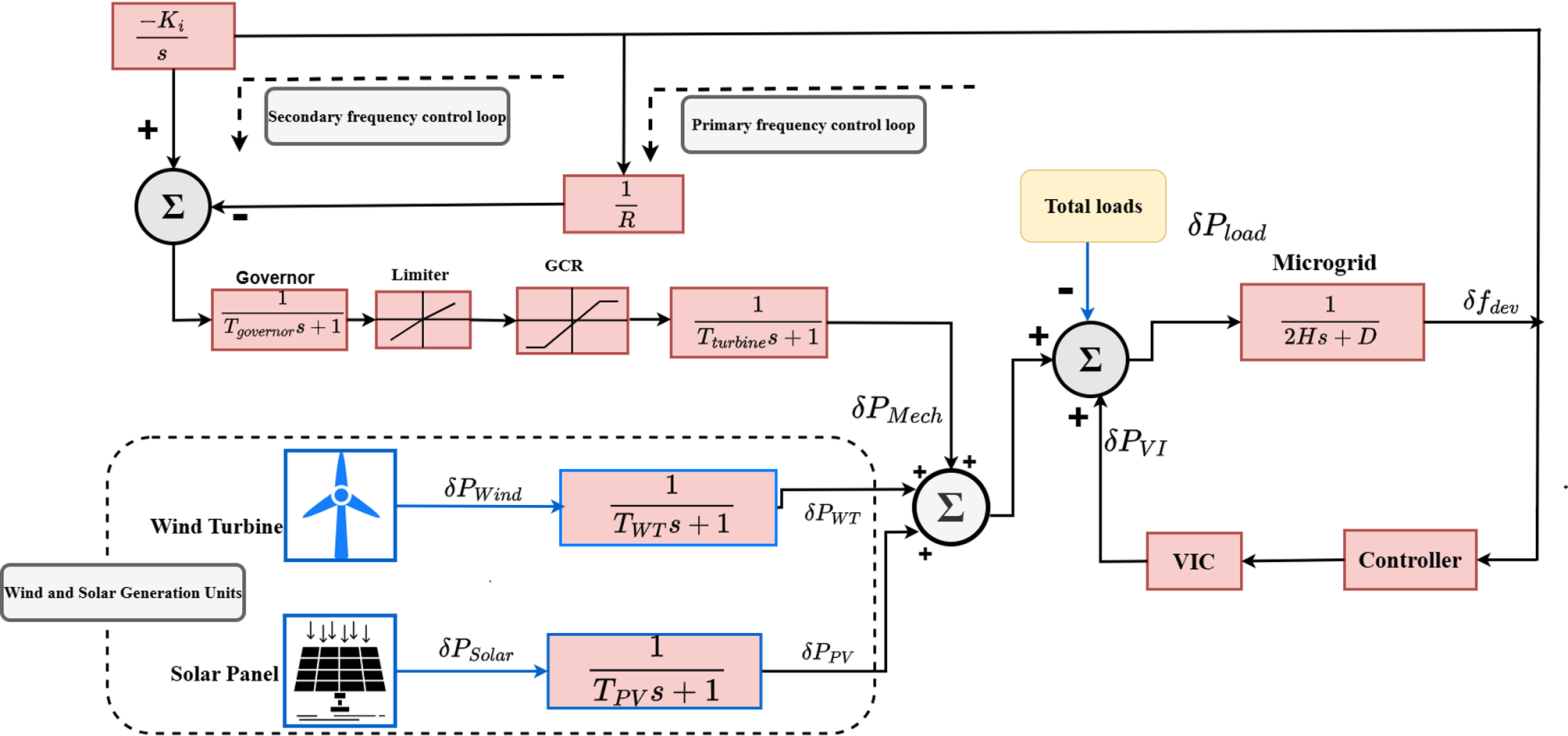
Dynamic model of the islanded microgrid considering high penetration of RESs. Generated using [draw.io] v29.6.10.

Islanded MG is composed of a variety of RESs, including solar, wind, and an energy storage system. It utilizes a sensor to measure the generated power and power quality assessment items, then transmits the measured values to an MG control system. Islanded MG system was used to assess the performance of the proposed controller which consisted of solar system, wind turbine system, energy storage system and thermal power plant as illustrates in Fig. 4 The model of islanded MG with the proposed controller illustrates in Fig. 5. Table 1 shows the disturbance ($\:\delta\:{P}_{Wind}$,$\:\:\delta\:{P}_{PV}$, and $\:\delta\:{P}_{L}$ ) and the physical constraints GCR and GBD are considered in order to provide a precise impression of islanded MG. Additionally, the opening and closing of the valve are restricted by the maximum and minimum limitations (VU, VL)^31^. The islanded MG frequency deviation taking into account the impact of secondary and main frequency control. The GRC for the non-reheat thermal power plant is specified as 20% p.u. MW/minute^31^. The dynamic structure of the system under study, considering the dynamic impacts of VIC with frequency measurement, is built as shown in Fig. 5 in order to perform the frequency stability analysis. Table 2 presents the MG parameters and all controller parameter values associated with the PID and PIDA controllers. This study’s low order dynamic model is sufficiently accurate to analyze frequency stability problems^29^.

**Table 1:** Summary of disturbances and physical constraints in islanded MG.

Symbol	Description	Type
$\:\delta\:{P}_{Wind}$	Wind power variation	Disturbance
$\:\delta\:{P}_{PV}$	Solar power variation	Disturbance
$\:\delta\:{P}_{L}$	Load power variation	Disturbance
GCR	Governor constraint rate	Physical constraint
GDB	Governor Dead-Band	Physical constraint
$\:{V}_{U}$	Upper valve limit	Saturation constraint
$\:{V}_{L}$	Lower valve limit	Saturation constraint

The frequency deviation of islanded MG could be described as follows^32,33^.

$$\:\delta\:f=\frac{1}{2Hs+D}(\delta\:{p}_{m}+\delta\:{p}_{WT}+\delta\:{p}_{PV}+\delta\:{p}_{inertia}-\delta\:{p}_{L})$$

$$\:\delta\:{p}_{m}=\left(\varDelta\:{p}_{g}\right)\times\:\frac{1}{{T}_{t}s+1}\:\:\:\:\:\:$$

$$\:{\delta\:p}_{g}=(\delta\:{p}_{c}-\frac{1}{R}\varDelta\:f)\times\:\frac{1}{{T}_{g}s+1}$$

$$\:\delta\:{p}_{WT}=\left(\delta\:{p}_{wind}\right)\times\:\frac{1}{{T}_{WT}s+1}$$

$$\:\delta\:{p}_{PV=}\left(\delta\:{p}_{solar}\right)\times\:\frac{1}{s{T}_{PV}+1}$$

Where, $\:\delta\:$f is the MG frequency deviation, $\:\delta\:{p}_{m}$is the mechanical power variation, $\:\delta\:{p}_{WT}$ and $\:\delta\:{p}_{PV}$ are the wind and PV power variation, $\:\delta\:{p}_{VI}$ is the VI power, $\:\delta\:{P}_{L}$ is the load disturbance and $\:\delta\:{P}_{g}$is the governor output power. The studied islanded MG is represented using a linearized small-signal model around the nominal operating point. The analysis focuses on active-power/frequency dynamics. The transfer function of the MG represents the frequency in the MG where H is the equivalent inertia constant, D is the damping coefficient. As shown in Fig. 5, the $\:\delta\:$f signal is processed by the controller, whose output is applied to the VI loop to generate the auxiliary inertia support signal. This signal is injected into the system to improve the transient frequency response.

## Virtual inertia-based control strategy and methodology

The frequency stability of an isolated MG is dependent on the inertia of the MG^34,35^. In order to improve the dynamic frequency stability, and to increase the penetration of RESs, dynamic frequency control faster than the primary frequency control of synchronous generator needs to be added. In other words, inertia can be set into the system through an energy storage system, such as a battery, an ultracapacitor, etc. A VI is defined as the combination of an energy storage system, a power electronics converter and proper control algorithm that improves the dynamic frequency stability of the system. In this research, the VI power is created by using energy storage system. The Laplace-based per-unit expression of the VI power emulation strategy is illustrated in Eq. (6).

During frequency deviation, the power required by the MG is supplied by the VIC system, as given in Eq. (6).

6 $$\:\delta\:{p}_{VI}=\frac{{K}_{VI}}{1+s{T}_{VI}}\left(\frac{d\delta\:f}{dt}\right)\:\:$$

Where, $\:{T}_{VI}$ is the time constant-based VI to emulate the dynamic control of the energy storage system in the studied MG, and $\:{K}_{VI}$ is the gain of VIC in the MG.

### Conventional controllers

PID and PIDA controllers were used in this study to enhance the frequency stability and reduce the frequency rate of change (ROCOF).

#### PID controller

**Fig. 6:**
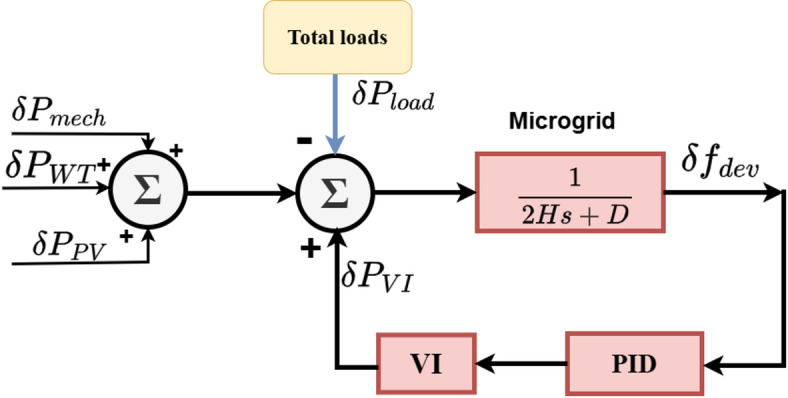
Virtual inertia control based PID. Generated using [draw.io] v29.6.10.

PID controllers are widely used in industry because of their relatively simple design and the adequate performance they may offer for a wide range of applications. The following variables can be used to assess the PID controller’s efficacy: the derivative gain ($\:{K}_{d}$), integral gain ($\:{K}_{i}$), and propositional gain ($\:{K}_{p}$)^36^. Figure 6 illustrates a PID scheme based on VIC. Equation (7) represents the PID controller formal in Laplace function. When there are strong control requirements in a particular application and the process has advanced dynamics PID controllers may not be able to provide the necessary performance due to their basic designs^37^. The PID controller was initially tuned using the MATLAB/Simulink PID Tuner tool, and then further refined through simulation-based fine tuning to obtain a satisfactory dynamic response under the considered operating conditions.

7 $$\:M\left(s\right)={K}_{p}+\frac{{K}_{i}}{s}+{K}_{d}s\:$$

#### PIDA controller

**Fig. 7:**
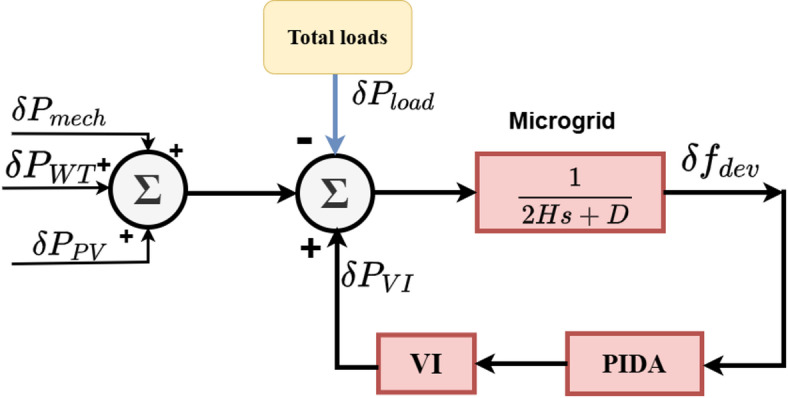
Virtual inertia control based PIDA. Generated using [draw.io] v29.6.10.

Because of their simple designs, PID controllers might not be able to provide the required performance. PIDA controllers are the subject of an increasing amount of research these days. The double derivative acceleration ($\:{K}_{a}$) of the control error to the traditional PID control law can result in significant performance improvements for high-order systems or integral processes^38^. Figure 7 illustrates a PIDA scheme based on the VIC model in Eq. (8), representing the PIDA controller in the Laplace domain.

18 $$\:M\left(s\right)=\frac{{K}_{a}{s}^{3}+{K}_{d}{s}^{2}+{K}_{p}s+{K}_{i}}{{s}^{3}+\mu\:{s}^{2}+\varPhi\:s}\:\:$$

Where$\:,\:\mu\:$ and $\:\varPhi\:$ are two new parameters. This controller’s different feature is acceleration by momentum acquisition, which improves the response’s stability limits by adding new roots to the characteristic’s equation. A manual tuning method in MATLAB/Simulink was used to acquire the PIDA gain Similar iterative procedures were used in the manual tuning phase, but the acceleration term was also tuned. To get a consistent response, the $\:{K}_{i}$, $\:{K}_{p}$, and $\:{K}_{d}$ gains were first modified. The dampening of quick transients was then improved by gradually increasing the $\:{K}_{a}$ gain. The relative weighting of derivative and acceleration actions was controlled by adjusting the parameters α and β. The system response was tracked at every stage of a number of MATLAB/Simulink trials, and the gains were adjusted to reduce maximum frequency deviation and steady-state error. When the best performance and stability were achieved across all disturbance conditions, the final tuning set was chosen.

The critical analysis which was presented in^39^ is used to determine the value of the PIDA parameter.

**Table 2:** Microgrid system and controller parameters.

Microgrid parameter	Notation	Value
**Microgrid Damping Coefficient**	𝐷	0.015
**Governor time constant**	$\:{\mathrm{T}}_{\mathrm{g}}$(s)	0.1
**Turbine time constant**	$\:{\mathrm{T}}_{\mathrm{t}}$(s)	0.4
**Wind turbine time constant**	$\:{\mathrm{T}}_{\mathrm{W}\mathrm{T}}$(s)	1.5
**Solar system time constant**	$\:{\mathrm{T}}_{\mathrm{P}\mathrm{V}}$(s)	1.8
**Speed droop characteristic**	R	2.4
**Integral control variable gain**	$\:{\mathrm{K}}_{i}$(s)	0.05
**Virtual inertia control gain**	$\:{\mathrm{K}}_{\mathrm{V}\mathrm{I}}$(s)	0.5
**Virtual inertia time constant**	$\:{\mathrm{T}}_{\mathrm{V}\mathrm{I}}$(s)	10
**Maximum limit of valve gate**	$\:{\:\:\mathrm{V}}_{U}$	0.3
**Minimum limit of valve gate**	$\:{\:\:\:\:\:\:\:\:\:\:\:\:\:\:\:\:\:\mathrm{V}}_{\mathrm{l}}$	-0.3
**Generation rate constraints**	GCR	20%
**Frequency**	F	50
**Microgrid system inertia**	H	0.083
**PID controller**	$\:{\mathrm{K}}_{\mathrm{p}}$	13
	$\:{K}_{i}$	15
	$\:{\mathrm{K}}_{\mathrm{d}}$	12
**PIDA controller**	$\:{\mathrm{K}}_{\mathrm{p}}$	296
	$\:{K}_{i}$	297
	$\:{\mathrm{K}}_{\mathrm{d}}$	200
	$\:\alpha\:$	15
	$\:\beta\:$	0.5

### Proposed intelligent controller

**Fig. 8:**
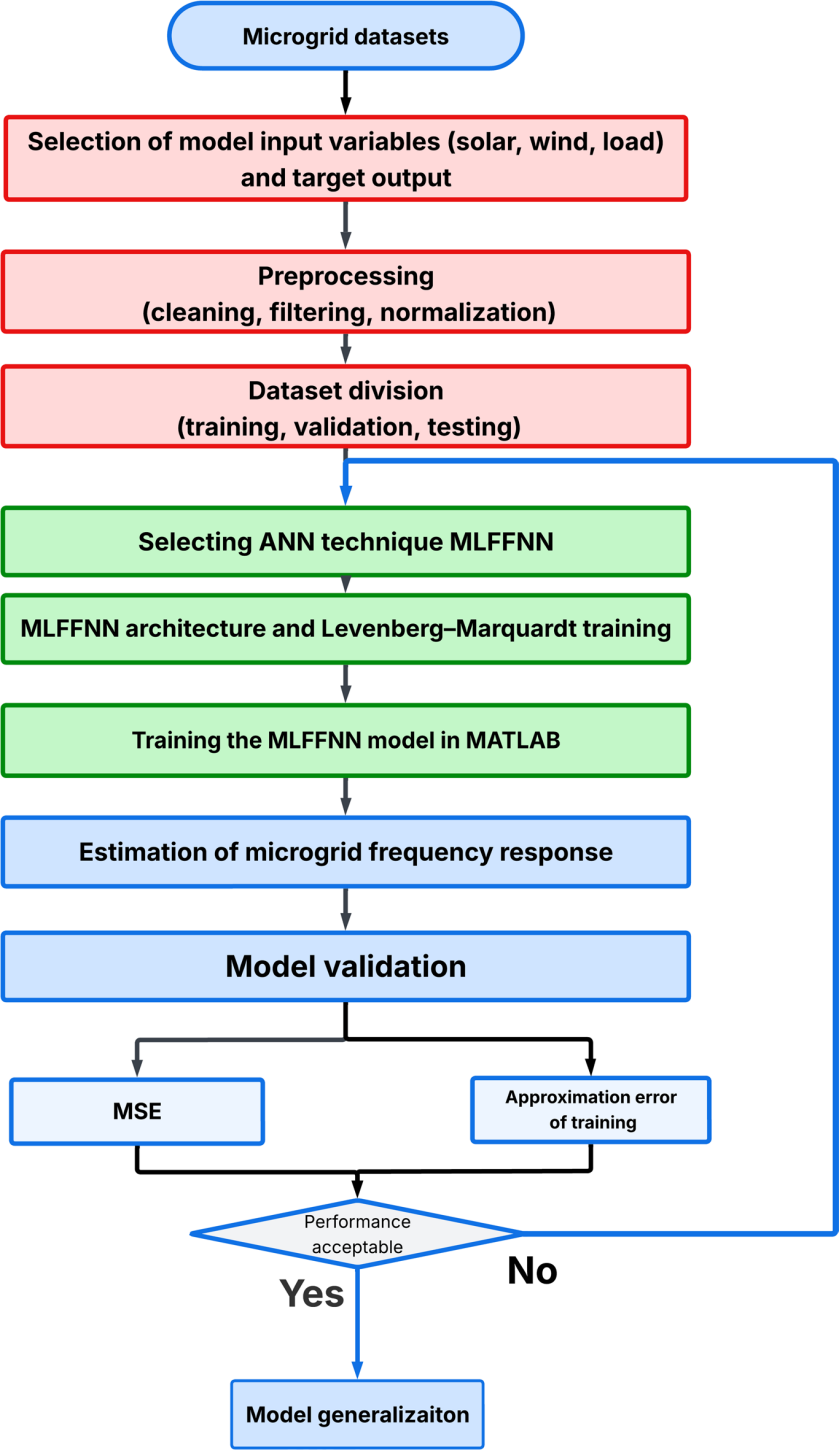
The proposed MLFFNN-based modeling and validation procedure for the islanded microgrid.

**Fig. 9:**
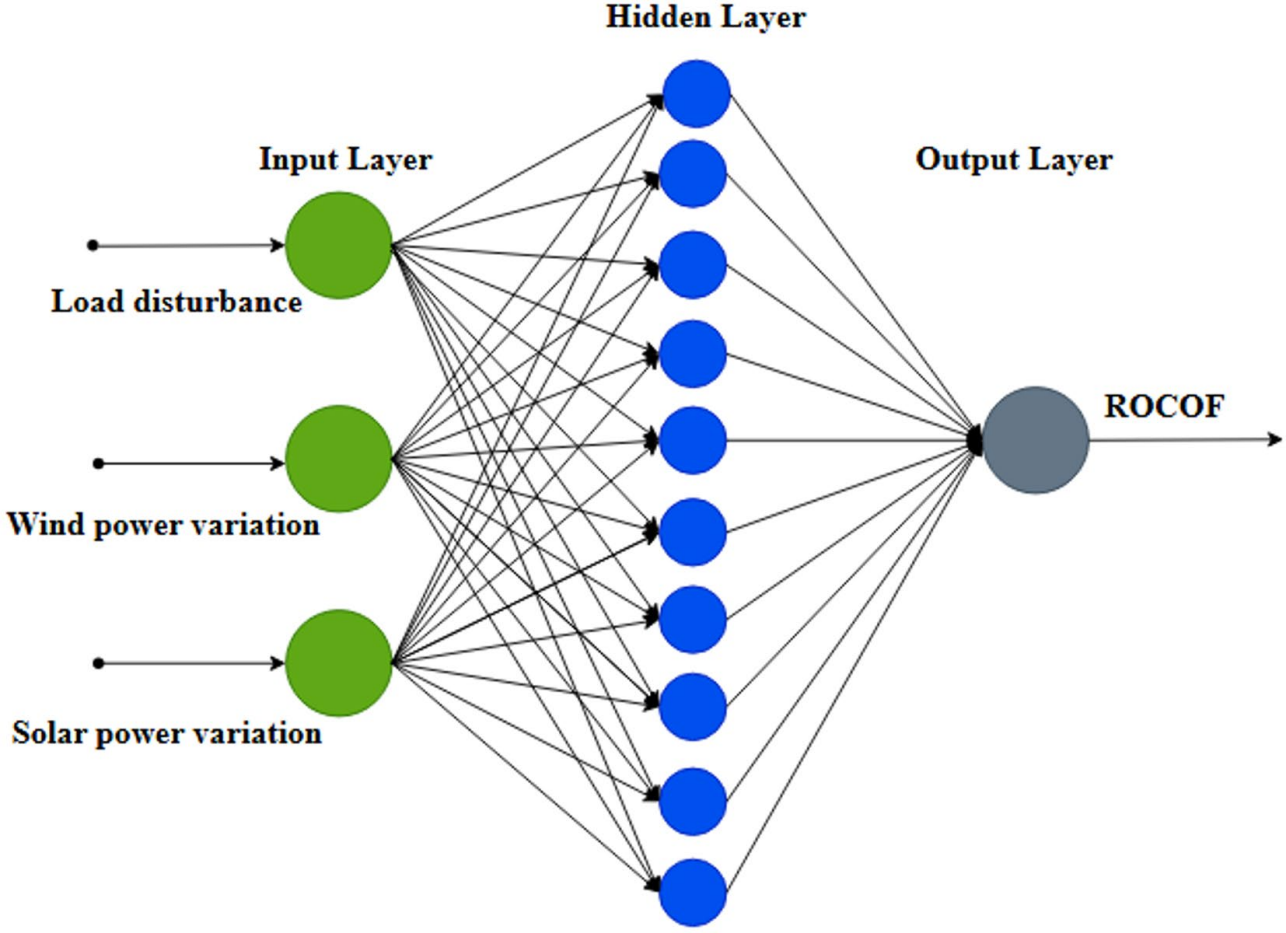
Architecture of the MLFFN. Generated using [draw.io] v29.6.10.

Because of the large number of parameters in the PIDA controller, its sensitivity to noise and the complexity of achieving optimal tuning parameters under different scenarios^40^, it is difficult to carry out in real-time applications because of its high computational burden^41^. Modelling the human reasoning process as a computationally efficient method is the main purpose of artificial neural networks. Multiple layers of artificial neurons make up the MLFFNN, which creates unidirectional input and output forward connections^42^. The suggested neural network algorithm, which fell into the following stages and was depicted in Fig. 8, was used to build the suggested technique. The stages include the following: dataset preparation, variable selection, preprocessing, dataset division, network configuration training and model validation. The MLFFNN design comprised three layers: the input layer, which stored load, solar power and wind power variation as its inputs; the hidden layer and output layer, which produced the frequency deviation as shown in Fig. 9^43^. The three-input signal were selected because they directly affect the system frequency dynamics. The network output represents the ROCOF, which is used as an indicator of the dynamic frequency response and for generating the appropriate control action. The dataset was generated offline using MATLAB/Simulink simulations of the studied islanded MG under different operating scenarios, including load changes, wind power variations, and PV power fluctuations. The MLFFNN is trained offline, and its weights remain fixed during online operation. In addition, the hidden-layer activation function is selected as tanh, which is bounded. Therefore, the generated neural-network control signal remains bounded. Since the MLFFNN acts as an auxiliary adaptive signal within the cascaded MLFFNN-VI structure, while the VI block provides the main inertial support, the proposed controller supports bounded closed-loop behaviour under the studied operating conditions and bounded disturbances.

Equation (9) shows the weight sum of MLFFNN inputs.

9 $$\:{m}_{j}=\:{Q}_{j}\left({\sum\:}_{j}^{3}{w}_{ji}{k}_{i}\right)$$

$\:{k}_{i}$ is the MLFFNN$\:{\prime\:}$s inputs$\:\:{k}_{1}=\delta\:{P}_{solar}$,$\:\:{k}_{2}=\varDelta\:{P}_{wind}$, $\:{k}_{3}=\delta\:{P}_{load}$ and w is the weight between input and hidden neurons.

10 $$\:{E}_{j}=\mathrm{t}\mathrm{a}\mathrm{n}\mathrm{h}\left({m}_{j}\right)$$

Equation (10) represents the activation function. The MLFFNN’s output is given by Eq. (11). The activation function tanh used due to its zero-centered output and bounded range, which help achieve balanced gradient updates and improve training stability^44^.

11 $$\:\delta\:f{\prime\:}=\mathrm{t}\mathrm{a}\mathrm{n}\mathrm{h}\left({\sum\:}_{j=0}^{n}{A}_{1j}{E}_{j}\right)$$

**Fig. 10:**
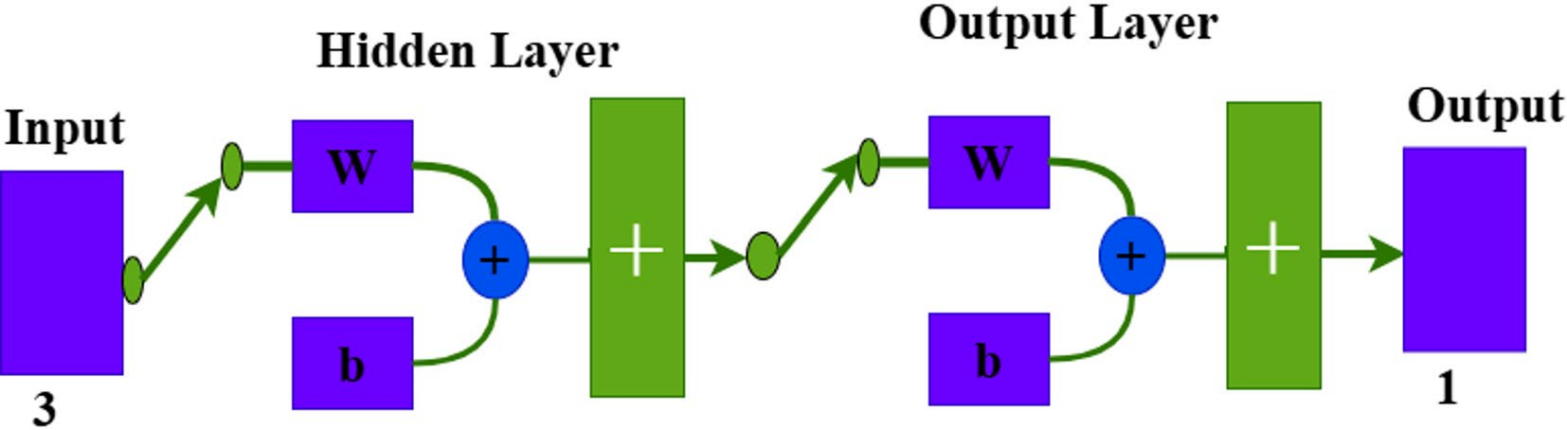
The structure of neural network form MATLAB-SIMULINK program.

Where,$\:\:{A}_{1j}$ is the weight between the hidden neurons j and $\:{\updelta\:}f{\prime\:}$ represents the ROCOF produced by MLFFNN. The proposed controller was designed with three inputs (load variation, solar and wind variation), a single hidden layer consists of 10 neurons which achieved the best performance among the evaluated network configuration, and one output which represents ROCOF. The network is shown in Fig. 10.

**Fig. 11:**
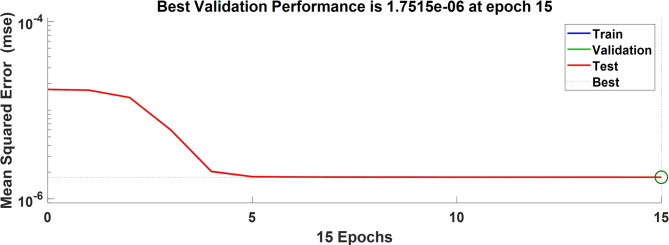
MSE of training the neural network MLFFNN.

The network was trained using data from load variation, solar and wind variation. The network was trained using the Levenberg-Marquardt backpropagation algorithm with mean square error (MSE) as the performance. The generated dataset includes different disturbance magnitudes and operating profiles in order to improve the diversity of the training samples. Moreover, to reduce overfitting, the dataset was divided into 70% of the dataset were used for training,15% used for validation, and 15% used for testing to ensure robustness. The maximum number of epochs was set to 1000 iterations. However, the training converged after 15 iterations, achieved $\:1.7515\times\:{10}^{-6}$ as shown in Fig. 11. Each controller provides specific strength and limitations depending on its control mechanism. Table 3 presents the advantages and disadvantages of the PID, PIDA, and MLFFNN controllers.

**Table 3:** Advantages and disadvantages of the PID, PIDA, and MLFFNN controllers.

PID	Advantages	• PID controllers are extensively adopted in industrial applications owing to their simple structure, while the presence of effective tuning rules further simplifies their design process^37,45^. • For a wide range of applications, PID controllers offer good performance^36,46^. • For first- and second-order systems, PID controllers provide good performance after parameter tuning^36^. • The ease of implementation and clear operational characteristics of PID controllers have contributed to their widespread industrial use^46^.
	**Disadvantages**	• Owing to their structural simplicity, PID controllers may be unable to meet stringent performance requirements, particularly under demanding control conditions^36,37^. • In higher-order systems, PID controller tuning becomes more challenging, and their effectiveness depends on the availability of accurate mathematical models^36^.
**PIDA**	**Advantages**	• Second derivative action improves PIDA performance in high-order dynamic system, have three zeros instead of two, which enhances their control capability and provide improved performance for high-order processes compared to conventional PID controller^37–39^.
	**Disadvantages**	• PIDA controllers introduce a slightly more complex structure compared to PID controllers.
**MLFFNN**	**Advantages**	• The MLFFNN can complete online training, enabling real-time adaptation and improved performance^47^.
		• Successful operation of MLFFNN in different scenarios^48^.
		• The flexible nonlinear connection between input and output^49^.
	**Disadvantages**	• This type of network requires several input targets during the training phase. Insufficient training data could impair network performance and raise the possibility of overfitting^43^.

**Fig. 12:**
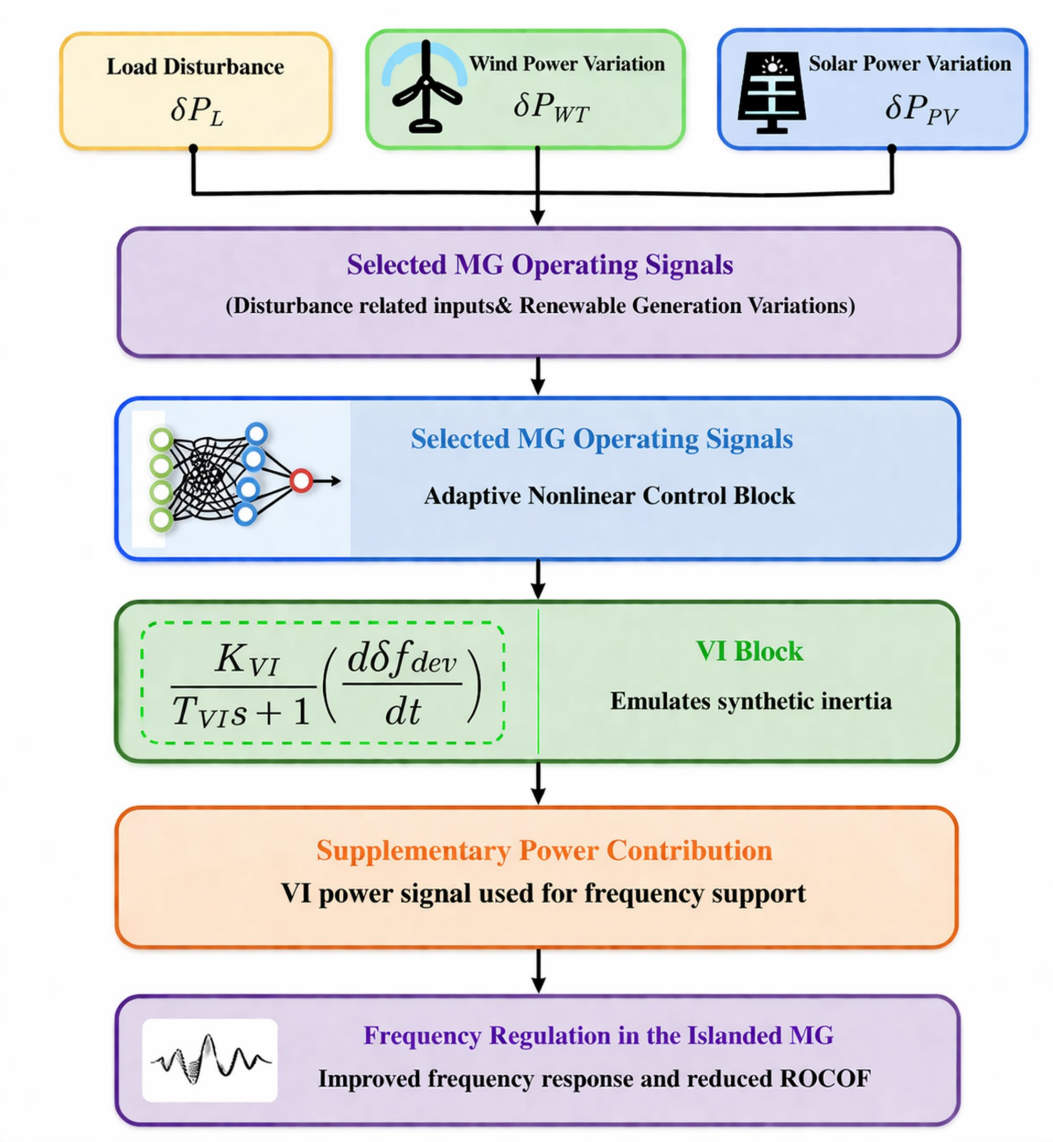
Proposed MLFFNN-VI control scheme for frequency regulation in the islanded MG.

The proposed controller is implemented as a hybrid control structure. First, the MLFFNN receives the selected MG operating signals, including disturbance-related inputs and renewable generation variations, and produces an adaptive control signal according to the system dynamic condition. Then, this signal is passed through the VI block to emulate synthetic inertia and generate the supplementary power signal required for frequency support. In this way, the MLFFNN improves the adaptability of the control action, while the VI block ensures fast inertial response during sudden disturbances. The proposed MLFFNN-VI controller combines the adaptive nonlinear action of the MLFFNN with the inertial support provided by the VI block. The VI contribution is determined according to Eq. (6), while the MLFFNN generates the supplementary signal based on the selected disturbance-related inputs. The overall control scheme of the proposed controller is shown in Fig. 12.

## Simulation results and discussion

The simulation results are used to assess the effectiveness of the proposed control strategy in maintaining the MG output frequency stable. The simulation results were simulated using MATLAB. The efficiency of the MG was studied with the proposed VIC -based PID, the PIDA and MLFFNN controller were checked and assessed. The MG and controller parameters used in this study are summarized for clarity and fair comparison given in Table 2^32,50,51^. A simulation was performed under three scenarios to demonstrate the suggested strategy’s efficacy by applying power variations pattern of wind and solar generation and a random load demand. The suggested controller was evaluated under the same load variation patterns in^31^ and another load variation. in the first scenario. In addition to the comparison case, the system was evaluated under two additional scenarios.

### SCENARIO I: applying step load change

**Fig. 13:**
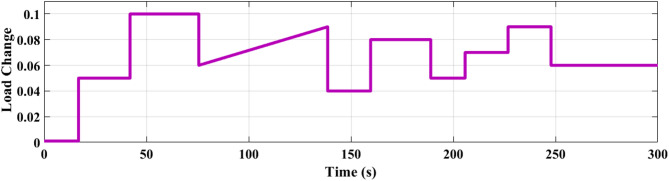
Power pattern under random load demand.

**Fig. 14:**
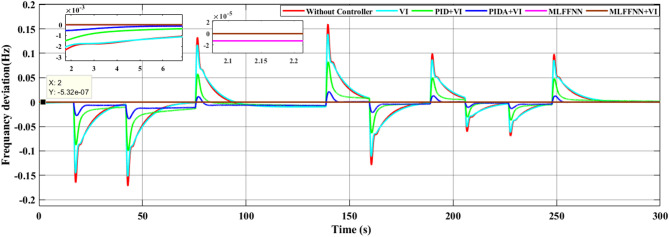
The microgrid frequency deviation under the load power variation.

A step load change was applied to MG, as shown in Fig. 13. In the first case, the performance of the studied MG with the proposed VIC based on PID, PIDA and MLFFNN controllers were tested and assessed by applying different load patterns only as shown in Fig. 14 which display the frequency deviation of the studied MG with the different control; the proposed VIC based PID, VIC -based PIDA, VIC, and without VIC.

**Fig. 15:**
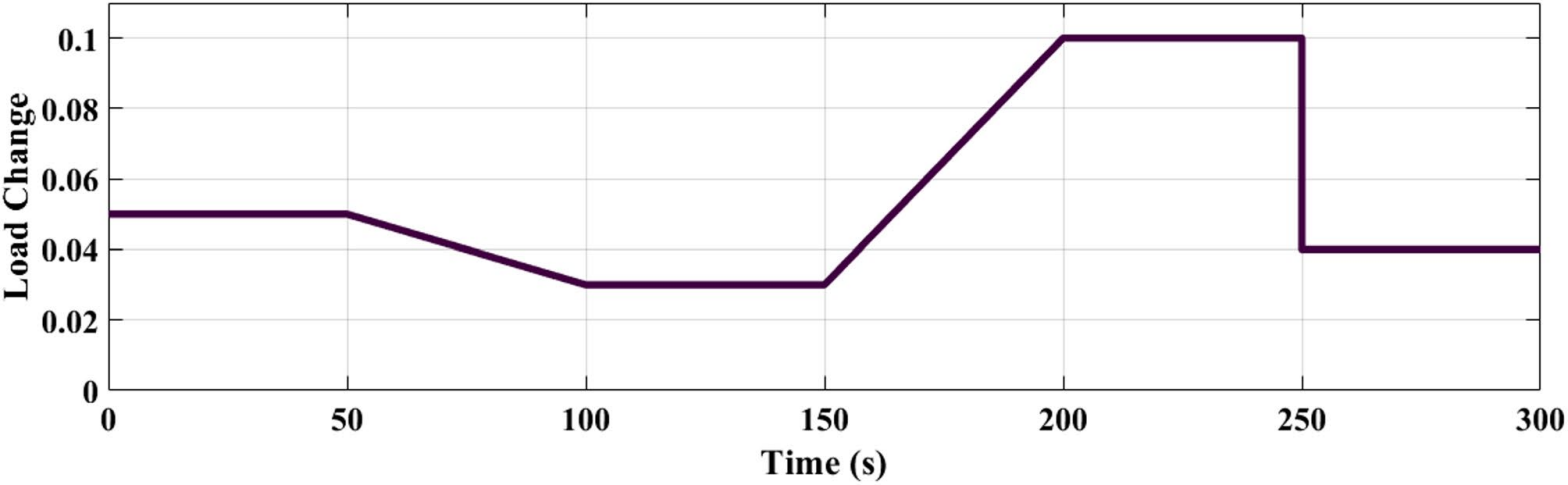
Power pattern under random load demand.

**Fig. 16:**
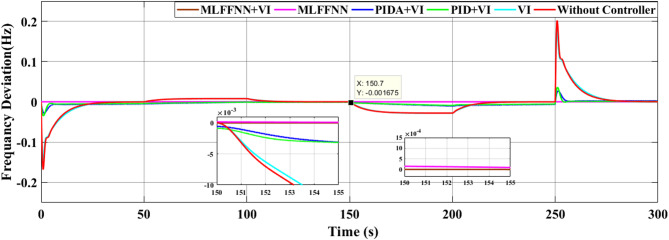
The microgrid frequency deviation under the load power variation.

Figure 14 shows the frequency deviation of the MG under load power variations. As shown in Fig. 14, the system without a controller exhibits the largest frequency deviations whenever a load disturbance occurs, with noticeable overshoot, undershoot peaks and a slow recovery back to the nominal value. Traditional controls VI, PID + VI, and PIDA + VI were applied, the frequency response improved compared to without any controller. However, these controllers still demonstrated relatively higher overshoots and longer settling times, especially during large and sudden load variations. The MLFFNN-based controller provided better effectiveness compared to other controllers, as it decreased the frequency deviation. Therefore, when combined with VI, the recommended controller demonstrated the highest efficiency. whereas the MLFFNN enhances the adaptive control action under changing operating conditions. When both components are combined, the resulting controller achieves the best dynamic frequency performance, which confirms that the improvement arises from their cooperative interaction rather than from either component alone. Out of all the disturbance events, the MLFFNN with VI showed the lowest frequency variation. The MLFFNN with VI controller achieved the frequency stability under load power changes. It was clear that the proposed controller improved the frequency stability of MG, reduced the maximum overshoot and settling time compared to conventional controls like PID, PIDA, and VI-based controllers. The controller’s performance was presented in Table 4 which represents the MSE, standard deviation (STD), minimum absolute error and maximum absolute error for each controller in this state. The results indicated that MLFFNN with VI achieved the lowest performance. Even MLFFNN without VI provided better performance compared to other controllers as shown in Table 4. The system also evaluated with another step load change as shown in Figs. 15 and 16; Table 5 present the performance of the different controllers under another step load change. Due to the stochastic and continuously varying nature of RES inputs, conventional indices such as settling time are less representative; therefore, additional performance indices were considered for a more reliable evaluation.

**Table 4:** Performance of different controllers in terms of MSE, STD, minimum absolute error and maximum absolute error.

Controller	MSE	STD	Minimum absolute error	Maximum absolute error
**Without VI**	0.001242	0.0350488	$\:7.6451{\times\:10}^{-3}$	0.1718385
**With VI**	0.0011277	0.033344	$\:4.5136{\times\:10}^{-3}$	0.1522902
**PID + VI**	3.154$\:{\times\:10}^{-4}$	0.017247	$\:3.811{\times\:10}^{-4}$	0.0995002
**PIDA + VI**	5.678$\:{\times\:10}^{-5}$	0.0062818	5.612$\:{\times\:10}^{-5}$	1.169$\:{\times\:10}^{-7}$
**MLFFNN**	9.307$\:{\times\:10}^{-10}$	2.591$\:{\times\:10}^{-5}$	4.0496$\:{\times\:10}^{-6}$	6.796$\:{\times\:10}^{-5}$
**MLFFNN + VI**	8.301$\:{\times\:10}^{-13}$	9.098$\:{\times\:10}^{-7}$	4.2692$\:{\times\:10}^{-11}$	4.839$\:{\times\:10}^{-6}$

**Table 5:** Performance of different controllers in terms of MSE, STD, minimum absolute error and maximum absolute error.

Controller	MSE	STD	Minimum absolute error	Maximum absolute error	Maximum frequency deviation) Hz)
**Without VI**	7.131$\:{\times\:10}^{-4}$	0.026506	0.0004514	0.202034	0.013668
**With VI**	6.705$\:{\times\:10}^{-4}$	0.02577	0.0003154	0.178288	0.0134355
**PID + VI**	3.142$\:{\times\:10}^{-5}$	0.004614	0.0000546	0.036268	0.00398
**PIDA + VI**	3.819$\:{\times\:10}^{-5}$	0.004959	0.0000046	0.02807	0.004818
**MLFFNN**	6.124$\:{\times\:10}^{-9}$	6.3443$\:{\times\:10}^{-5}$	1.961$\:{\times\:10}^{-7}$	1.508$\:{\times\:10}^{-4}$	5.8134$\:{\times\:10}^{-5}$
**MLFFNN + VI**	1.4257$\:{\times\:10}^{-12}$	1.1895$\:{\times\:10}^{-6}$	6.6844$\:{\times\:10}^{-10}$	4.2641$\:{\times\:10}^{-6}$	7.028$\:{\times\:10}^{-7}$

As shown in Table 4, the proposed MLFFNN + VI controller achieved the lowest values for all reported error indices among the compared controllers. In particular, the MSE decreased from 0.0011277 for VI to 8.301$\:{\times\:10}^{-13}$ for MLFFNN + VI, while the STD was reduced from 0.033344 to 9.098$\:{\times\:10}^{-7}\:$. Moreover, the maximum absolute error dropped from 0.1522902 to 4.839$\:{\times\:10}^{-6}$, confirming the better regulation accuracy and oscillation suppression capability of the proposed hybrid controller. Although the proposed MLFFNN-VI controller is primarily validated through time-domain simulation, the obtained closed-loop responses under different disturbance scenarios confirm stable operation, improved damping, and the absence of sustained oscillations. This indicates that, the proposed controller preserves acceptable dynamic stability while enhancing frequency regulation performance.

### SCENARIO II: random wind and solar variation

**Fig. 17:**
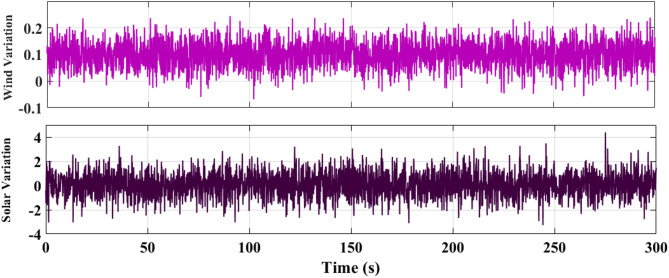
Power variations pattern of wind and solar generations.

**Fig. 18:**
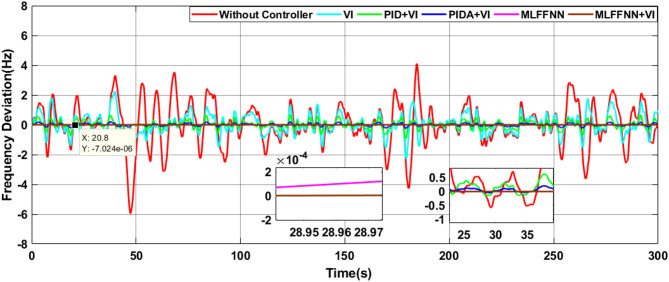
The microgrid frequency deviation under RESs variations.

In the second case, wind and solar variation were applied to MG as shown in Fig. 17. The performance of the studied MG with the proposed VIC -based on PID, PIDA and MLFFNN controllers was tested and assessed by applying random solar and wind patterns as shown in Fig. 18. The proposed controller MLFFNN with VI improves the stability of the MG under RESs variation, improved maximum overshoot. compared to conventional control like PID, PIDA, and VI-based controllers. The controller’s performance as shown in Table 6 which represented MSE, STD, minimum absolute error and maximum absolute error for each controller in this state. The results showed that MLFFNN with VI achieved the lowest performance. Even MLFFNN without VI provided better performance compared to other controllers as shown in Table 5.

**Table 6:** Performance of different controllers in terms of MSE, STD, minimum absolute error and maximum absolute error.

Controller	MSE	STD	Minimum absolute error	Maximum absolute error
**Without VI**	2.0735054	1.4394773	$\:3.6487{\times\:10}^{-3}$	5.96135995
**With VI**	0.4681744	0.6836983	$\:3.6571{\times\:10}^{-3}$	2.27142319
**PID + VI**	0.0667299	0.25774319	$\:6.8231{\times\:10}^{-4}$	0.916715
**PIDA + VI**	0.0068026	0.0816046	2.541$\:{\times\:10}^{-6}$	0.23294223
**MLFFNN**	5.102$\:{\times\:10}^{-8}$	2.227$\:{\times\:10}^{-4}$	8.6529$\:{\times\:10}^{-8}$	9.398$\:{\times\:10}^{-4}$
**MLFFNN + VI**	1.239$\:{\times\:10}^{-10}$	1.113$\:{\times\:10}^{-5}$	4.778$\:{\times\:10}^{-9}$	4.5416$\:{\times\:10}^{-5}$

Figure 18 shows the frequency deviation of the MG under RESs. As shown in Fig. 18, the system without a controller exhibits the largest frequency deviations whenever a large oscillation occurred. Traditional controls VI, PID + VI, and PIDA + VI were applied, the frequency response became more stable compared to without any controller. Although these controllers reduce the magnitude of oscillations, they still show moderate fluctuations due to the rapid and unpredictable changes in RES generation. The MLFFNN-based controller provided better performance by decreased the oscillation amplitude and enhancing the smoothness of the frequency response compared to other controllers, as it decreased the frequency deviation. So, the suggested controller showed the best efficiency when combined with VI. The MLFFNN with VI demonstrated the smallest frequency deviation and the fastest settling time across all disturbance events. The MLFFNN with VI controller achieved the frequency stability under RESs fluctuation.

As shown in Table 6, the proposed MLFFNN + VI controller achieved the lowest values for all reported error indices among the compared controllers. In particular, the MSE decreased from 0.0068026 for PIDA + VI to 1.239$\:{\times\:10}^{-10}$ for MLFFNN + VI, while the STD was reduced from 0.0816046 to 1.113$\:{\times\:10}^{-5}\:$. Moreover, the maximum absolute error dropped from 0.23294223 to 4.5416$\:{\times\:10}^{-5}$, confirming the better regulation accuracy and oscillation suppression capability of the proposed hybrid controller.

### SCENARIO III: load and RES variations

**Fig. 19:**
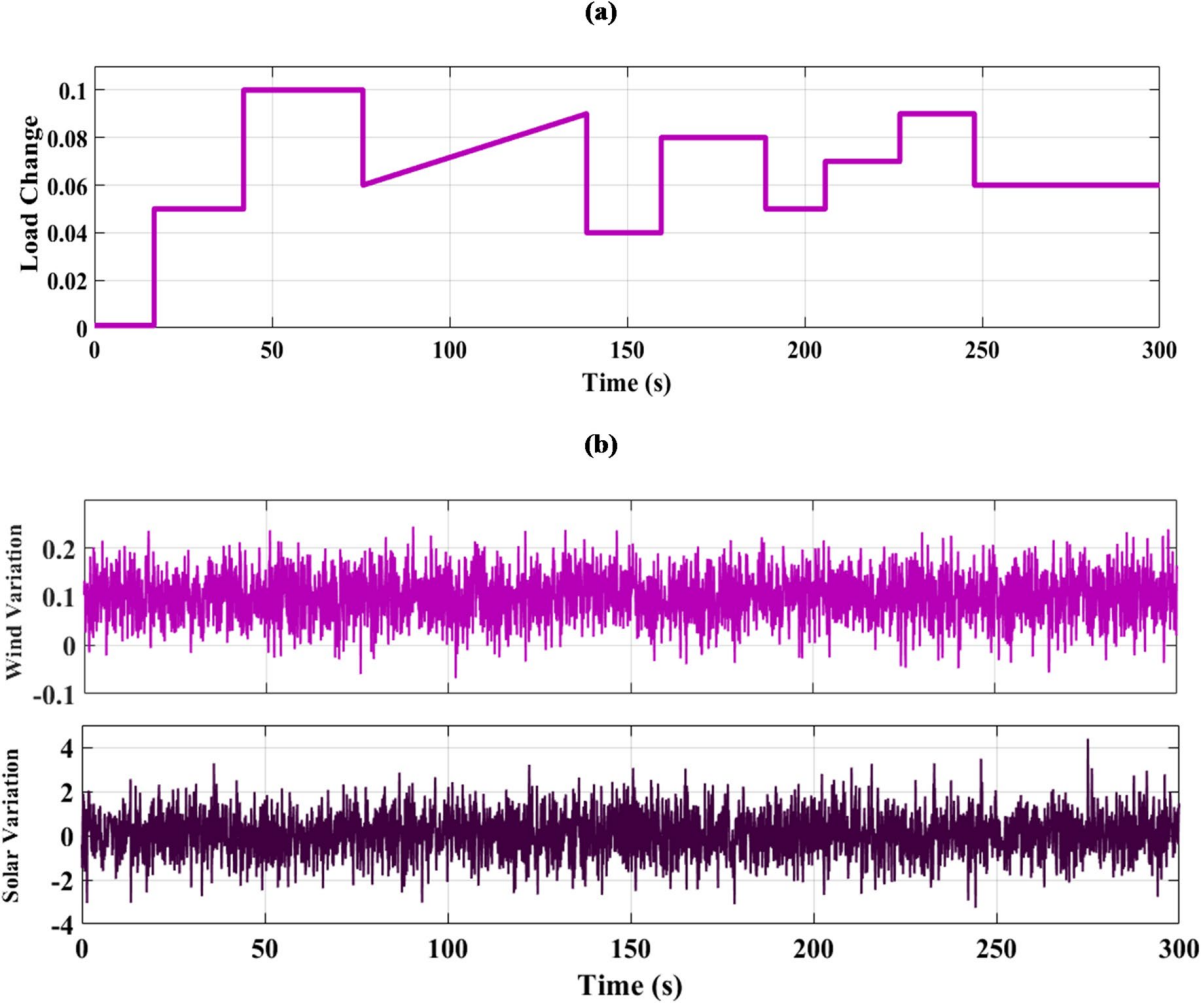
(**a**) Power pattern under random load demand. (**b**) Wind and solar power variations.

**Fig. 20:**
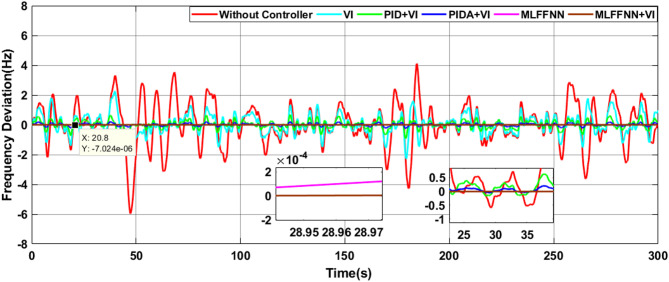
The MG frequency deviation under both variation of load power and RESs.

Step load change and RESs variation were applied to MG as shown in Fig. 19 The performance of the studied MG with the proposed VIC -based on PID, PIDA and MLFFNN controllers was tested and evaluated by applying random solar and wind patterns as shown in Fig. 20. The proposed controller MLFFNN with VI improved the stability of the MG, improved maximum overshoot under both load variation and RESs. Compared to conventional controls like PID, PIDA, and VI-based controllers, the controller’s performance as shown in Table 7 which represented MSE, STD, minimum absolute error and maximum absolute error for each controller in this state. The results show that MLFFNN with VI achieved the lowest performance. Even MLFFNN without VI provided better performance compared to other controllers as shown in Table 7.

**Table 7:** Performance of different controllers in terms of MSE, STD, minimum absolute error and maximum absolute error.

Controller	MSE	STD	Minimum absolute error	Maximum absolute error
**Without VI**	1.57577628	1.25470673	$\:1.2648{\times\:10}^{-2}$	4.9202029
**With VI**	0.3834707	0.61874119	$\:7.6971{\times\:10}^{-3}$	2.318265
**PID + VI**	0.0639167	0.25254193	$\:2.5176{\times\:10}^{-4}$	0.9005055
**PIDA + VI**	0.00688789	0.08263557	$\:3.8114{\times\:10}^{-5}$	0.24663068
**MLFFNN**	4.158$\:{\times\:10}^{-8}$	2.021021$\:{\times\:10}^{-4}$	4.431$\:{\times\:10}^{-8}$	7.969$\:{\times\:10}^{-4}$
**MLFFNN + VI**	1.011$\:{\times\:10}^{-10}$	1.005$\:{\times\:10}^{-5}$	3.571$\:{\times\:10}^{-9}$	4.106$\:{\times\:10}^{-5}$

The frequency deviation of the MG under both load and RES variation is shown in Fig. 20. Every time there is a significant oscillation, the uncontrolled system showed the largest frequency variations. When conventional controls VI, PID + VI, and PIDA + VI were used, the frequency response stabilized more than when no controller was used. Due to the quick and unpredictable changes in RES generation and load variation, these controllers exhibit considerable fluctuations even though they lessen the oscillations’ magnitude. Compared to previous controllers, the MLFFNN-based controller performed better by reducing the oscillation amplitude and improving the frequency response’s smoothness by lowering the frequency deviation. Therefore, when paired with VI, the recommended controller demonstrated the highest efficiency. The MLFFNN with VI demonstrated the smallest frequency deviation across all disturbance events. The MLFFNN with the VI controller achieved frequency stability under RESs fluctuation and load variation.

As shown in Table 7, the proposed MLFFNN + VI controller achieved the lowest values for all reported error indices among the compared controllers. In particular, the MSE decreased from 0.00688789 for PIDA + VI to 1.011 × 10⁻¹⁰ for MLFFNN + VI, while the STD was reduced from 0.08263557 to 1.005 × 10⁻⁵. Moreover, the maximum absolute error dropped from 0.24663068 to 4.106 × 10⁻⁵, confirming the better regulation accuracy and oscillation suppression capability of the proposed hybrid controller.

### Generalization performance evaluation

**Fig. 21:**
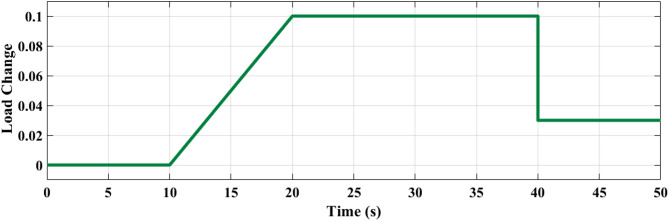
Power pattern under random load demand.

**Fig. 22:**
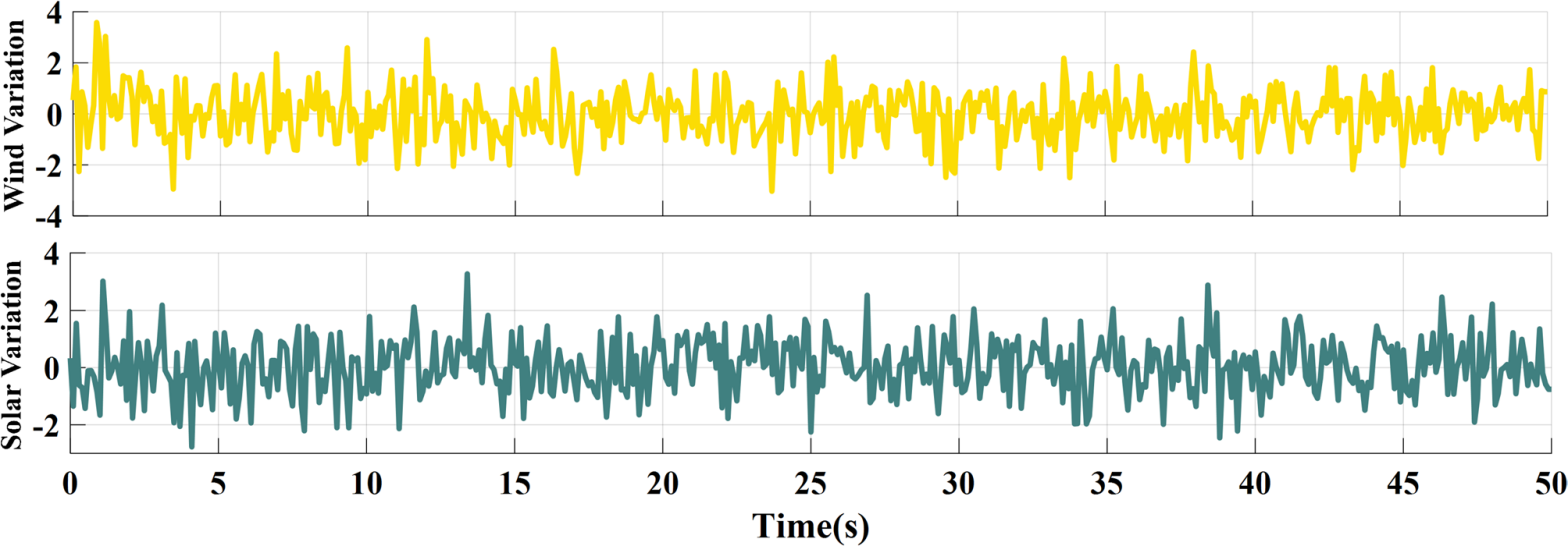
Power variations pattern of wind and solar generations.

**Fig. 23:**
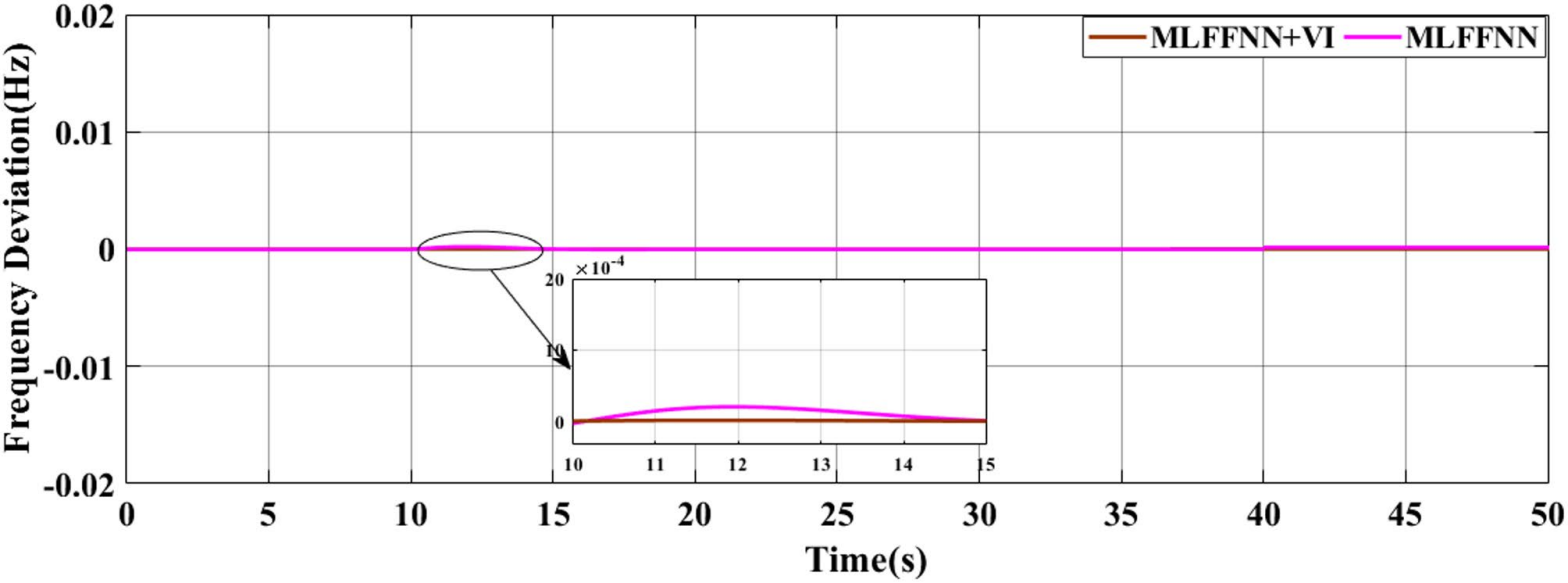
The microgrid frequency deviation under the load power variation.

**Fig. 24:**
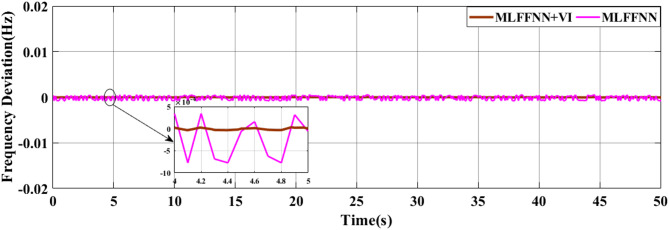
The microgrid frequency deviation under RES variation.

**Fig. 25:**
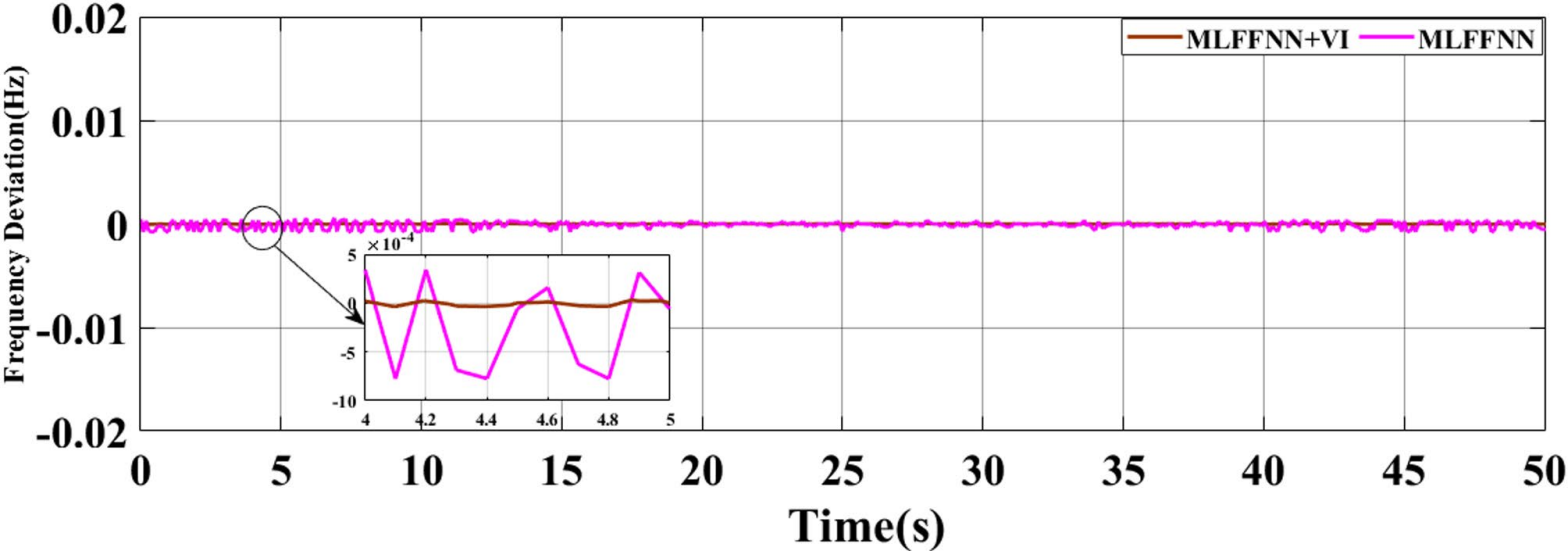
The microgrid frequency deviation under both RES variation and load change.

To maintain the frequency regulation performance when the system is subjected to new operating conditions that weren’t included during the training process is known as generalization, so a new step change load and RES load were applied to the system to evaluate the performance of the suggested controller, as shown in Figs. 21 and 22 respectively. The system was studied under three scenarios. In the first scenario, a step change load was only applied to MG as shown in Fig. 23 and the controller’s performance as shown in Table 8. The performance of the MG system was studied with the MLFFNN. In the second scenario wind and solar variation were applied as shown in Fig. 24 and the controller’s performance as shown in Table 9. In the last case RESs and step load change were applied to evaluate the performance of the studied controller as shown in Fig. 25 and the controller’s performance as shown in Table 10. The proposed controller enhances the stability of the MG under RESs variation and step load change. As shown in Figs. 23 and 24.25, the proposed controller achieved the best performance with reduced overshoot, when VI was integrated with the proposed controller, which improved further, particularly in reducing the ROCOF due to the ability of VI to emulate the inertial behavior of synchronous generator.

**Table 8:** Performance comparison of different controllers under step load change.

Controller	Maximum frequency deviation )Hz)	STD	MSE	Minimum absolute error	Maximum absolute error
**MLFFNN + VI**	2.139$\:{\times\:10}^{-6}$	3.089$\:{\times\:10}^{-6}$	1.028$\:{\times\:10}^{-11}$	0	9.834$\:{\times\:10}^{-6}$
**MLFFNN**	6.067$\:{\times\:10}^{-5}$	7.967$\:{\times\:10}^{-5}$	7.214$\:{\times\:10}^{-9}$	4.064$\:{\times\:10}^{-7}$	1.993$\:{\times\:10}^{-4}$

**Table 9:** Performance comparison of different controllers under RES variation.

Controller	Maximum frequency deviation (Hz)	STD	MSE	Minimum absolute error	Maximum absolute error
**MLFFNN + VI**	2.187$\:{\times\:10}^{-5}$	2.379$\:{\times\:10}^{-5}$	5.696$\:{\times\:10}^{-10}$	0	3.865$\:{\times\:10}^{-5}$
**MLFFNN**	4.607$\:{\times\:10}^{-4}$	4.885$\:{\times\:10}^{-4}$	2.671$\:{\times\:10}^{-7}$	9.012$\:{\times\:10}^{-7}$	7.756$\:{\times\:10}^{-4}$

**Table 10:** Performance comparison of different controllers under both RES variation and load change.

Controller	Maximum frequency deviation (Hz)	STD	MSE	Minimum absolute error	Maximum absolute error
**MLFFNN + VI**	1.308$\:{\times\:10}^{-5}$	1.667$\:{\times\:10}^{-5}$	2.785$\:{\times\:10}^{-10}$	0	3.865$\:{\times\:10}^{-5}$
**MLFFNN**	2.784$\:{\times\:10}^{-4}$	3.441$\:{\times\:10}^{-4}$	1.314$\:{\times\:10}^{-7}$	2.306$\:{\times\:10}^{-7}$	7.756$\:{\times\:10}^{-4}$

**Fig. 26:**
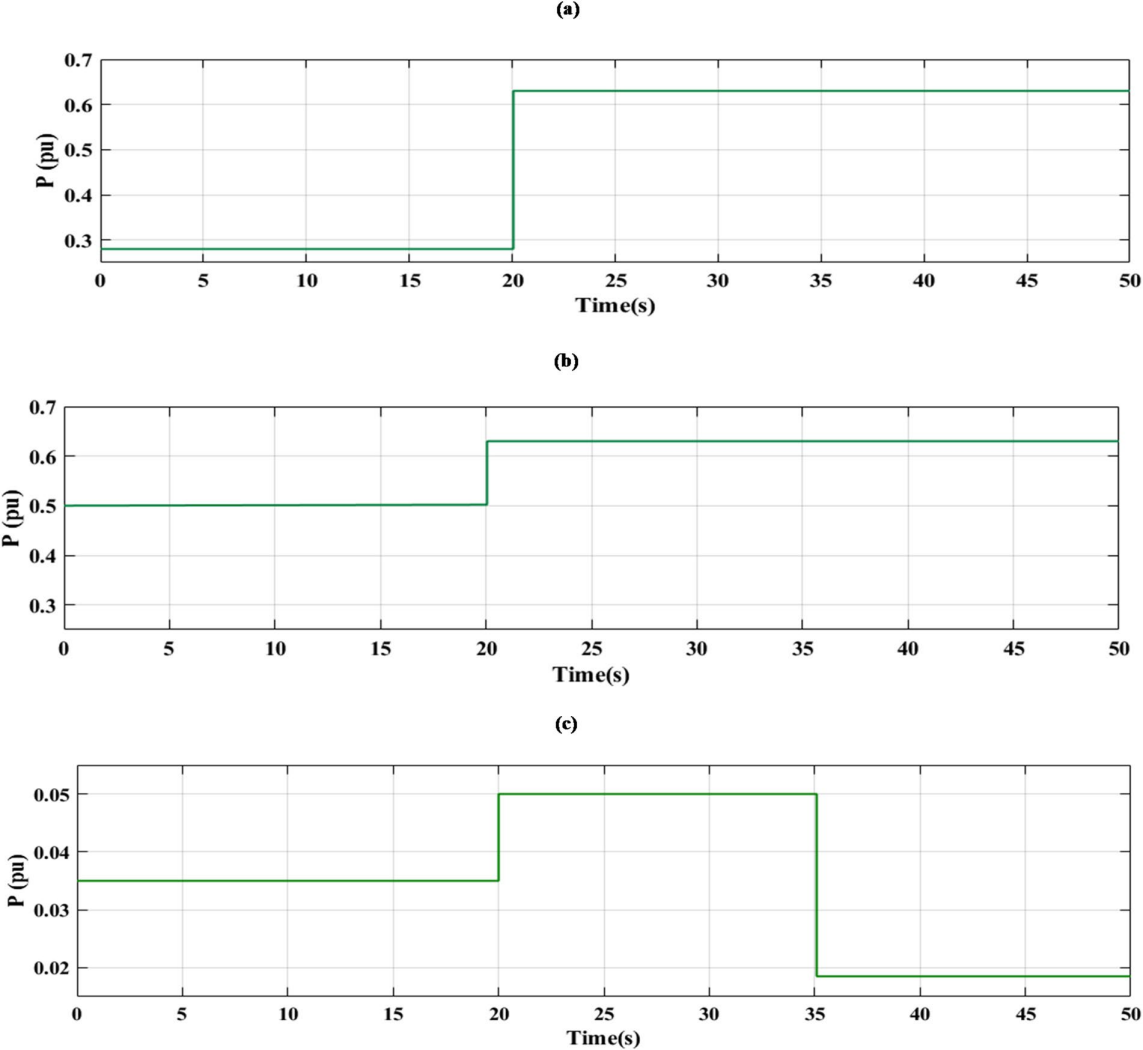
Considered input load disturbance scenarios for generalization assessment: (**a**) moderate step increase, (**b**) large step increase, and (**c**) double-step variation.

**Fig. 27:**
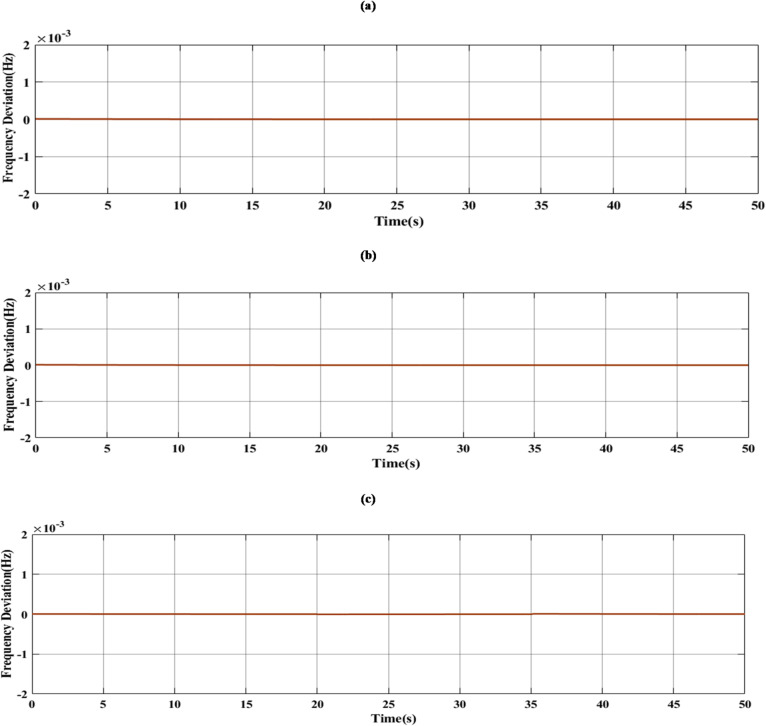
Corresponding output frequency deviation responses of the proposed MLFFNN + VI controller under the considered load disturbance scenarios: (a) moderate step increase, (b) large step increase, and (c) double-step variation.

Additional disturbances were applied to examine the generalization capability beyond the main simulation cases. The result proved that the proposed controller maintained stable and improved frequency regulation performance under new operating conditions. indicting that the proposed controller is not limited to single disturbance pattern. The input disturbance scenarios are illustrated in Fig. 26 (a)–(c), while the corresponding output responses are shown in Fig. 27 (a)–(c). Table 11 summarizes the performance of the proposed MLFFNN + VI controller under the three considered load disturbance scenarios, together with the corresponding mean values of the reported indices. Table 12 presents a comparative analysis between the results in this study and recent works from literature. The suggested controller achieved the lowest frequency deviation across all disturbance scenarios. To enable a comprehensive comparison, this study extends the existing literature by extracting and evaluating multiple performance indicators that were not explicitly reported in previous works. Although the achieved deviation in the controlled case is very small, this result reflects the adopted simulation assumptions and ideal model conditions. In practical MG systems, larger frequency deviations may arise due to measurement noise, communication delays, converter dynamics, parameter uncertainty, and energy storage constraints.

**Table 11:** Performance evaluation of the proposed MLFFNN + VI controller under three load disturbance scenarios along with the corresponding mean values.

Disturbance scenario	Controller	Maximum frequency deviation (Hz)	STD	MSE	Minimum absolute error	Maximum absolute error
**Moderate load step increase at t = 20 s**	**MLFFNN + VI**	1.9309$\:{\times\:10}^{-6}$	$2.5206\:{\times\:10}^{-6}$	$1.0077\:{\times\:10}^{-11}$	0	1.1041$\:{\times\:10}^{-5}$
**Large load step increase at t = 20 s**		1.934$\:{\times\:10}^{-6}$	$2.5037\:{\times\:10}^{-6}$	$1.0004\:{\times\:10}^{-11}$	0	1.099$\:{\times\:10}^{-5}$
**Double step load variation at t = 20 s and t = 35 s**		2.9944$\:{\times\:10}^{-6}$	$3.2815\:{\times\:10}^{-6}$	$1.1806\:{\times\:10}^{-11}$	0	8.744$\:{\times\:10}^{-6}$
**Mean**		2.2864$\:{\times\:10}^{-6}$	$2.7686\:{\times\:10}^{-6}$	$1.0629\:{\times\:10}^{-11}$	0	1.0258$\:{\times\:10}^{-5}$

**Table 12:** Comparative performance between the proposed controller and recent microgrid studies under different disturbance scenarios.

Paper	Year	Controller	Analysis/ Generalization	No. inputs	MSE	STD	Minimum absolute error	Maximum absolute error	Maximum frequency deviation(Hz)	Integral absolute error
Proposed methods	2025	MLFFNN + VI	True	One input (Load variation)	$\:8.3015\times\:{10}^{-13}$	$\:9.0981\times\:{10}^{-7}$	4.2692$\:{\times\:10}^{-11}$	4.8398$\:{\times\:10}^{-6}$	4.839$\:{\times\:10}^{-6}$	$4.8649\:{\times\:10}^{-5}$
				Two inputs RESs variation	$\:1.2395\times\:{10}^{-10}$	$\:1.1132\times\:{10}^{-5}$	4.7780$\:{\times\:10}^{-9}$	4.5416$\:{\times\:\mathrm{x}10}^{-5}$	3.8653$\:{\times\:10}^{-5}$	0.0022
				Three inputs (Load, wind and solar variation)	$\:1.0114\times\:{10}^{-10}$	$\:1.0057\times\:{10}^{-5}$	3.5704$\:{\times\:10}^{-9}$	4.1066$\:{\times\:10}^{-5}$	3.8647$\:{\times\:10}^{-5}$	$9.4343\:{\times\:10}^{-4}$
		MLFFNN		One input (Load variation)	9.3071$\:{\times\:10}^{-10}$	2.5906$\:{\times\:10}^{-5}$	4.0496$\:{\times\:10}^{-6}$	6.7968$\:{\times\:10}^{-5}$	6.7968$\:{\times\:10}^{-5}$	0.00264
				Two inputs RESs variation	5.1023$\:{\times\:10}^{-8}$	2.2217$\:{\times\:10}^{-4}$	8.6529$\:{\times\:10}^{-8}$	9.3981$\:{\times\:10}^{-4}$	7.7562$\:{\times\:10}^{-4}$	0.04499
				Three inputs (Load, wind and solar variation)	4.1585$\:{\times\:10}^{-8}$	2.021$\:{\times\:10}^{-4}$	4.4302$\:{\times\:10}^{-8}$	7.9698$\:{\times\:10}^{-4}$	0.0008	0.0201
		PIDA + VI		One input (Load variation)	5.6788$\:{\times\:10}^{-5}$	0.0062818	5.612$\:{\times\:10}^{-5}$	1.1696$\:{\times\:10}^{-7}$	0.0343	0.5346
				Two inputs RESs variation	0.0068026	0.0816046	2.541$\:{\times\:10}^{-6}$	0.23294223	0.2329	6.6368
				Three inputs (Load, wind and solar variation)	0.00688789	0.08263557	$\:3.8114$ $\:{\times\:10}^{-5}$	0.24663068	0.2466	6.6751
		PID + VI		One input (Load variation)	3.15349$\:{\times\:10}^{-4}$	0.017247	$\:3.811$ $\:{\times\:10}^{-4}$	0.0995002	0.0995	1.1044
				Two inputs RESs variation	0.0667299	0.25774319	$\:6.8231$ $\:{\times\:10}^{-4}$	0.916715	0.9167	20.5528
				Three inputs (Load, wind and solar variation)	0.0639167	0.25254193	$\:2.5176$ $\:{\times\:10}^{-4}$	0.9005055	0.9005	20.1412
		With VI		One input (Load variation)	0.0011277	0.033344	$\:4.5136$ $\:{\times\:10}^{-3}$	0.1522902	0.1523	2.1630
				Two inputs RESs variation	0.4681744	0.6836983	$\:3.6571$ $\:{\times\:10}^{-3}$	2.27142319	2.2714	53.1064
				Three inputs (Load, wind and solar variation)	0.3834707	0.61874119	$\:7.6971$ $\:{\times\:10}^{-3}$	2.318265	2.3183	47.5979
		Without Controller		One input (Load variation)	0.001242	0.0350488	$\:7.6451$ $\:{\times\:10}^{-3}$	0.1718385	0.1718	0.1718
				Two inputs RESs variation	2.0735054	1.4394773	$\:3.6487$ $\:{\times\:10}^{-3}$	5.96135995	5.9614	107.8584
				Three inputs (Load, wind and solar variation)	1.57577628	1.25470673	$\:1.2648$ $\:{\times\:10}^{-2}$	4.9202029	4.9202	86.6345
Farhad et al. [15].	2025	VIC on PV, ESS, WT	False	(Load disturbance, Load + parameter uncertainty, load + RES disturbance)	Not reported	Not reported	Not reported	Not reported	(0.10, 0.12, 0.05)	Not reported
		VIC (PV + ESS)							(0.13 ,0.16, 0.16)	
		VIC (ESS only)							(0.17 ,0.21, 0.22)	
		No VIC							(0.22, 0.24, 0.19)	
Hamanah et al. [10].	2024	Improved VI controller	False	Constant load + RES	Not reported	Not reported	Not reported	Not reported	0.004	Not reported
				Unit Step load + RES					0.004	
				Radom load + RES					0.01	
Yegon et al. [5].	2023	PID + Adaptive Virtual Inertia (PSO/GA tuned)	False	Load disturbance (2%) **at t = 0.2 s**	For PSO-PID = 0.04846 ,For GA-PID = 0.1399	Not reported	Not reported	Not reported	1	Not reported
				Load disturbance (3%) **at t = 2.2 s**					1.5	
				Load disturbance (4%) at t = 4.5 s					2	
Khosravi et al. [26].	2020	FOPID	False	(Wind variation, solar variation, load fluctuations, all disturbance, all disturbance +uncertainty)	Not reported	Not reported	Not reported	Not reported	(0.15,0.015,0.1,0.1,0.12)	Not reported
H.Ali et al. [31].	2019	Combined VIC + MPC	False	Load variation	Not reported	Not reported	Not reported	Not reported	0.003	Not reported
				RESs Uncertainties					0.005	
				Severe Case with Parameter Uncertainties					0.002	
				RESs Uncertainty + Severe Parameter Variation					0	

## Conclusion

**This paper** presented a unique theory of frequency control based on virtual inertia control in order to sustain the frequency control loops of an insulated microgrid considering the high penetration rate of renewable energy sources. In addition, the proposed virtual inertia controller was first developed based on a simplified control strategy, namely PIDA and PID controller. To enhance the control accuracy and robustness, a MLFFNN controller was introduced and compared with the traditional control. The results show that MLFFNN has the best performance compared with PIDA and PID. The system was studied in three cases; in the first case, the microgrid system was evaluated by applying different load patterns only. In the second case, it was evaluated by applying the variating patterns of wind and solar power only. In the third case, it was evaluated by applying the variating patterns of wind and solar and different load patterns. Subsequently, the performance of the proposed virtual inertia control-based MLFFNN controller is compared with PIDA and PID under a variety of the RESs and system parameters variations to confirm the validation of the proposed controller. The results of the simulation were carried out using MATLAB software. Consequently, the results showed the excellent effectiveness of the proposed virtual inertia control-based MLFFNN controller compared to the traditional PIDA and PID controller. The MLFFNN does not inherently capture temporal dynamics, as it lacks an internal memory mechanism to model long-term time dependencies. Future work will address the practical implementation aspects, including computational burden and robustness under noise or communication delays, in addition to more challenging operating scenarios such as faults, abrupt islanding events, and parameter uncertainties.

## Data Availability

All data generated or analyzed during this study are included in this published article.

## Abbreviations

RES: Renewable energy sources
MGs: Microgrids
MLFFNN: Multi-layer feedforward neural network
VI: Virtual inertia
PID: Proportional-Integral-Derivative
PIDA: Proportional-Integral-Derivative-Acceleration
VIC: Virtual inertia control
DER: Distributed energy resources
PVs: Photovoltaics
EMS: Energy management system
FOPID: fractional-order PID
MSE: Mean square error
STD: Standard deviation
